# SGLT2 Inhibitors and the Risk of Infections in Type 2 Diabetes: Systematic Review and Meta-Analyses of Real-World Evidence

**DOI:** 10.1155/jdr/5888495

**Published:** 2025-10-16

**Authors:** Maria J. Alfonso Arvez, George S. Q. Tan, Miriam T. Y. Leung, Zanfina Ademi, J. Simon Bell

**Affiliations:** Monash Institute of Pharmaceutical Sciences, Centre for Medicine Use and Safety, Parkville, Australia

**Keywords:** COVID-19, infection, observational study, pneumonia, sepsis, SGLT2 inhibitor, Type 2 diabetes

## Abstract

**Background:**

People with diabetes are at increased risk of infections. Emerging evidence suggests sodium–glucose cotransporter 2 (SGLT2) inhibitors have pleiotropic effects that may protect against certain infections. We systematically reviewed real-world evidence on the association between SGLT2 inhibitors and infections among adults with Type 2 diabetes.

**Methods:**

We searched Medline, Embase, Scopus, and Google Scholar from January 1, 2012 to March 18, 2024 for observational studies conducted in adults with Type 2 diabetes published in English. The exposure was SGLT2 inhibitors, and comparators were nonusers or users of other glucose-lowering medications. Studies reporting outcome estimates for specific non-genitourinary infections were included. The study was prospectively registered with PROSPERO (CRD42023492265).

**Results:**

From 6827 records, 28 studies were included in qualitative synthesis and 14 in meta-analyses. There was no association with COVID-19-related mortality in seven studies (OR 0.91; 95% CI: 0.57–1.46) or COVID-19-related hospitalisation in three studies (OR 0.90; 95% CI: 0.67–1.20). A reduced risk of pneumonia was observed in three studies (HR: 0.61; 95% CI: 0.57–0.66), a reduced risk of pneumonia-related mortality in two studies (HR: 0.49; 95% CI: 0.35–0.67), and a reduced risk of sepsis in three studies (HR: 0.45; 95% CI: 0.30–0.68).

**Conclusion:**

Real-world evidence suggests SGLT2 inhibitors are associated with lower risk of pneumonia, pneumonia-related mortality and sepsis. Given the high burden of infection in this population, these associations deserve further research.

## 1. Introduction

Diabetes mellitus is a group of metabolic disorders characterised by hyperglycaemia. The prevalence of diabetes among adults is projected to increase to 693 million by 2045 [1]. Type 2 diabetes is the most common form and accounts for 90% of all diabetes cases globally [2]. In addition to well-known cardiovascular and renal complications, people with diabetes are at increased risk of infections [3]. Several factors contribute to this increased susceptibility, including the effects of hyperglycaemia on immune function, vascular insufficiency, neuropathy, skin barrier dysfunction and alterations in the gut microbiome [4]. Compared with the general population, individuals with diabetes have a higher risk of developing infections such as foot infections, respiratory tract infections, urinary tract infections (UTI), sepsis and postoperative infection [3]. Infections also contribute substantially to healthcare costs, with diabetes-related infections accounting for more than 48 billion dollars in hospitalisation costs in the United States alone [5].

Randomised controlled trials (RCTs) have reported mixed results regarding the association between SGLT2 inhibitors and different infections. Some RCTs have reported an increased risk of genitourinary infections, whilst others have suggested reductions in infection-related hospitalisations and lower respiratory tract infections [6–8]. Systematic reviews of RCTs have consistently shown an increased risk of genital infections but not UTIs [9, 10]. However, high-dose, long-term and add-on therapy with dapagliflozin has been associated with a higher risk of UTIs [11]. In 2015, the US Food and Drug Administration issued warnings about serious UTIs associated with SGLT2 inhibitors [12].

Evidence from observational studies is important to complement these findings. Many RCTs of SGLT2 inhibitors have excluded patients with a history of infections and have enrolled relatively small sample sizes, ranging from 71 to 7028 participants, with an average follow-up of only 42 weeks [13]. In contrast, well-conducted observational studies provide valuable evidence on long-term, patient-centred outcomes not routinely assessed in RCTs [14]. Several observational studies have specifically examined infections such as pneumonia and sepsis, which have not been comprehensively evaluated in RCTs, and some suggest that SGLT2 inhibitors may reduce the risk of these infections compared to other glucose-lowering medications [15, 16]. Therefore, the objective of this systematic review and meta-analyses was to critically evaluate real-world evidence on the association between SGLT2 inhibitors and infections among adults with Type 2 diabetes.

## 2. Materials and Methods

The protocol of this study was prospectively registered in the International Prospective Register of Systematic Reviews (PROSPERO) (ID: CRD42023492265).

### 2.1. Search Strategy and Databases

We searched Medline, Embase and Scopus to identify observational studies in English language published from 1 January 2012 to 18 March 2024. The date range was selected to coincide with the date of first licensing of SGLT2 inhibitors (dapagliflozin in the European Union by the European Medicines Agency) in 2012. Database search strategies were developed in consultation with a medical librarian. The search strategy incorporated terms such as (‘sodium glucose' OR ‘transporter 2 inhibitor∗' OR sglt∗) AND (‘observational studies' OR observational OR ‘clinical practice' OR ‘real world' OR ‘cohort stud∗' OR ‘case control') AND (effect∗ OR ‘patient safety' OR safety OR ‘drug related side effects' OR ‘adverse reactions' OR infection∗ OR outcome) (Supporting Information 1). Bibliometric database searches were supplemented by searching the first 10 pages of Google Scholar (first 100 articles organised by relevance). Reference and citation list of retrieved full text articles were manually searched to identify any additional potentially eligible studies. Articles referenced by systematic reviews or meta-analyses but not identified by the initial search were also included if they met the inclusion criteria. We contacted the authors of eligible studies to request or clarify data if necessary.

### 2.2. Eligibility Criteria

Studies were considered eligible for inclusion if they were conducted in adults with ype 2 diabetes (aged > 18 years), examined exposure to SGLT2 inhibitors (either as a class or individual agents), included a comparator group of nonusers of SGLT2 inhibitors or users of other glucose-lowering medications, reported infection-related outcomes with relative risk (RR), odds ratio (OR), and/or hazard ratio (HR) estimates, and used observational study designs (cohort or case-control). RCTs, literature reviews, conference abstracts, and commentaries were excluded. RCTs were excluded as this review aims to summarise evidence from real-world settings reported in observational studies. Studies without a comparison group, such as case series or case reports, were also excluded as it was not possible to extract risk estimates. Descriptive studies of spontaneous adverse event reports were excluded for the same reason. Studies conducted in patients with type 1 diabetes mellitus or where data for diabetes populations were not stratified by diabetes type were excluded. Furthermore, studies that did not report infection-related outcomes, reported infections as an overall outcome without specifying the type of infection, or focused solely on genitourinary infections were also excluded. The association between SGLT2 inhibitors and the risk of genitourinary infections has already been extensively studied and addressed in prior systematic reviews, allowing this analysis to focus on other types of infections [9, 10, 13, 17].

After removing duplicate records, two authors (M.J.A.A. and G.S.Q.T.) independently screened all titles and abstracts to determine potential eligibility. Discrepancies were resolved through discussion, with input from a third author (J.S.B.) when necessary. Full-text versions of articles identified during this screening phase were subsequently examined and evaluated for inclusion. Record screening was conducted using Covidence, a web-based tool designed for conducting systematic reviews.

### 2.3. Data Extraction

Data extraction was conducted independently by two authors (M.J.A.A. and G.S.Q.T.) using a predefined data extraction form. Discrepancies were resolved through discussion. Data extracted included bibliographic details (author and year), study characteristics (country, sample size and source of data), methodological aspects (design and duration of the study), treatment and comparator medication and patient participant demographics (age and sex). We also extracted the study outcomes for infection types including but not limited to respiratory tract infections (e.g., pneumonia) and gastrointestinal infections (e.g., gastroenteritis). It also included infection-related outcomes related to COVID-19 such as mortality and hospitalisation. All infection-related outcomes were extracted as defined in the original articles, and methods used to identify infections were summarised for each study. Finally, we extracted the outcome estimates for the infection outcomes as reported in the original studies, including OR, RR or HR, along with their corresponding 95% confidence intervals (CIs). These outcome estimates were reported descriptively, and no conversions between effect estimates were performed. We categorised studies by infection outcome, which meant some studies were listed more than once.

### 2.4. Risk of Bias Assessment

The Risk of Bias in Nonrandomised Studies of Interventions (ROBINS-I) tool was used to assess the methodological quality of included studies [18]. Two authors (M.J.A.A. and G.S.Q.T.) independently conducted the quality assessment. Any discrepancies were resolved through discussion between the two authors until consensus was reached. The ROBINS-I tool assists in identifying potential biases in estimating the comparative effectiveness of interventions, particularly in studies where participants were not randomised to different treatment groups. The tool emulates a hypothetical pragmatic randomised trial and evaluates seven domains where bias might arise: (1) confounding, (2) participant selection, (3) classification of interventions, (4) deviations from intended interventions, (5) missing data, (6) outcome measurement and (7) selection of the reported result. ROBINS-I utilises “signalling questions” to evaluate the risk of bias within each domain, with judgements in each domain contributing to an overall assessment of bias across all domains for the outcome under investigation. The overall risk of bias for a study was categorised as low, moderate, serious, or critical based on the highest risk of bias within any domain for the specific outcome of interest [19].

### 2.5. Statistical Analysis

Where possible we conducted meta-analyses for each infection-related outcome. We calculated pooled outcome estimates and the corresponding 95% CI only when at least two studies reported the same infection-related outcome [20]. Random effects models were used to account for between-study heterogeneity inherent in observational studies [21]. The pooled estimate for each infection-related outcome was estimated using the inverse of the logit function (inverse variance). We pooled available data on April 8, 2024. Forest plots were used to display the outcome estimate for the included studies and the pooled estimate for each infection-related outcome. We computed Higgin's and Thompson's *I*^2^ index to summarise heterogeneity across studies and *T*^2^ to estimate between-study variance under random-effects models [22]. *I*^2^ values of 25%, 50%, and 75% were considered to represent low, moderate, and high heterogeneity, respectively. For sensitivity analysis, we conducted a “leave-one-out analysis,” removing one study at a time from the analyses to determine if any single study exerted a disproportionate influence [20]. Publication bias was assessed by visually inspecting funnel plots of the accumulated evidence as well as quantitatively with Egger's regression [23]. A two-sided *p* value of < 0.05 was considered statistically significant. All statistical analyses were performed using RevMan Web, Version 7.7.2, and RStudio 2022.07.2.

The systematic review is reported in accordance with the Preferred Reporting Items for Systematic Review and Meta-Analysis (PRISMA) and Meta-analysis of Observational Studies in Epidemiology (MOOSE) guidelines (Supporting Information 2 and 3 for PRISMA and MOOSE checklists, respectively) [24, 25]

## 3. Results

### 3.1. Literature Search and Characteristics of Included Studies

**Figure 1:**
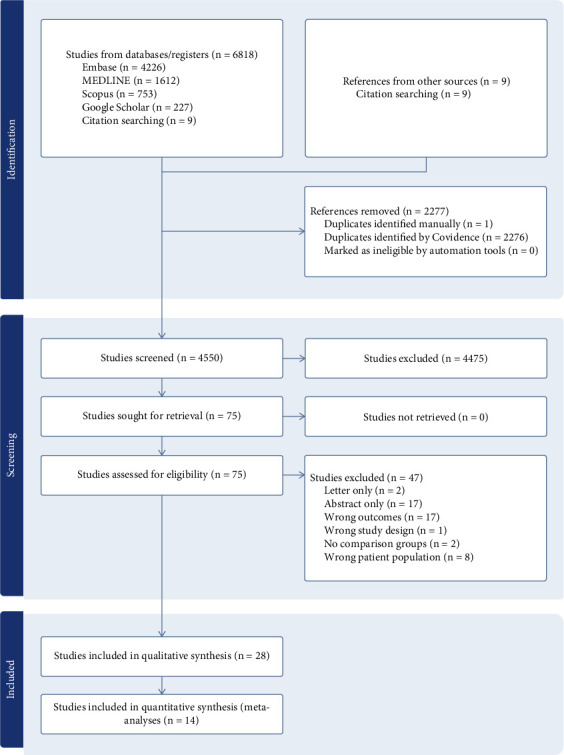
Study selection diagram.

A total of 6827 studies were identified through database searches up to March 18, 2024. After removing 2277 duplicates, 4550 remained. Following screening of abstracts and titles, 75 articles underwent full-text evaluation (Figure 1). Subsequently, 47 studies were excluded, including 17 abstracts, 17 with nonspecific infection outcomes, 8 not conducted in adults with Type 2 diabetes, 2 letters, 2 without a comparison group, and 1 cross-sectional study (Supporting Information 4). Finally, 28 studies met the eligibility criteria for qualitative synthesis, among which 14 studies were included for quantitative synthesis (meta-analysis). Studies were excluded from the meta-analysis either because they were the only study reporting a specific infection-related outcome, or because other studies reporting the same outcome used different effect estimates that could not be pooled. Of the 28 included studies, 25 investigated infections as the primary outcome, and three as a secondary outcome. Twenty-six were retrospective cohort studies, one was a prospective cohort study [26], and one was a nested case-control study [27]. Six studies were from the United Kingdom, four from Hong Kong and the United States of America, three from Italy, South Korea, and Spain, two from Taiwan, and one each from Sweden, Russia, and Australia (Table 1, Supporting Information 5).

### 3.2. Risk of Bias

**Figure 2:**
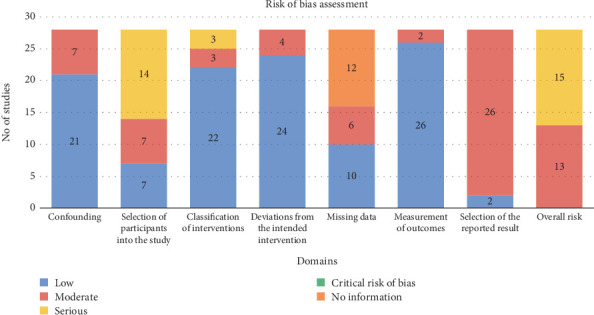
Risk of bias across studies using risk of bias in nonrandomised studies of interventions (ROBINS-I) tool.

The overall risk of bias was moderate in 13 studies and serious in 15 studies (Figure 2). Whilst none of the 28 included studies was deemed to have critical risk of bias overall, no study was deemed to have the lowest level risk of bias. Participant selection was the most common source of serious bias due to the inclusion of prevalent rather than incident users of the medications. Twelve studies did not discuss how missing data were handled and therefore were classified as “no information” for that domain. Selection of the reported result was moderate in 26 studies as they did not preregister study protocols specifying infection as an outcome. The remaining four ROBINS-I domains were all judged to be at low risk of bias. A detailed list of risk of bias assessments is provided in the Supporting Information 6.

### 3.3. Synthesis of Outcomes

#### 3.3.1. COVID-19 Mortality

**Figure 3:**
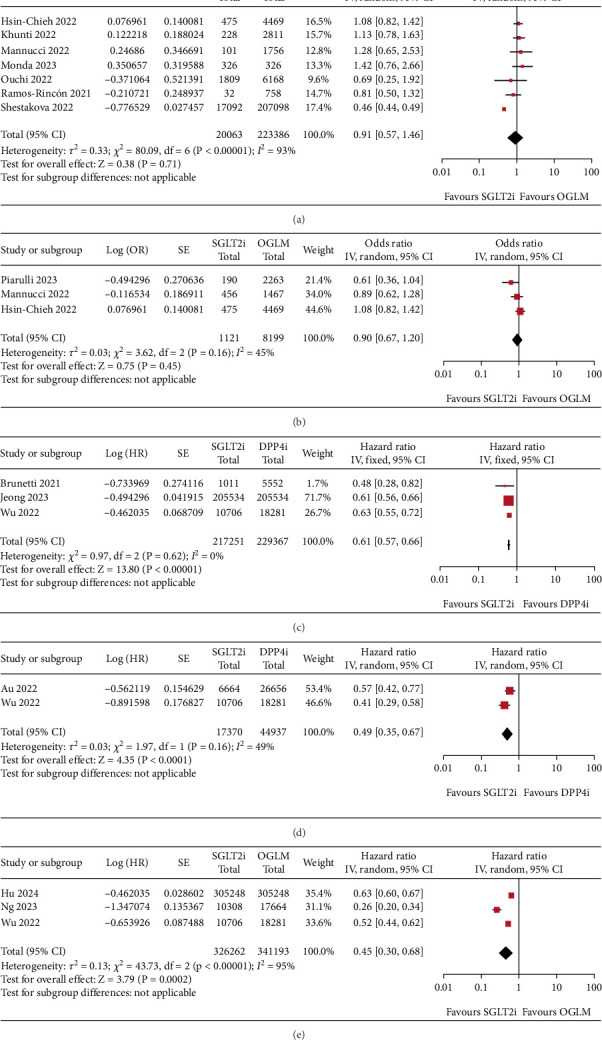
Forest plots that demonstrate the association between sodium–glucose cotransporter-2 inhibitors (SGLT2i) use with (a) COVID-19 mortality, (b) COVID-19 hospitalisation, (c) pneumonia, (d) pneumonia-mortality, and (e) sepsis in patients with Type 2 diabetes compared with other glucose-lowering medication (OGLM) or to dipeptidyl peptidase-4 inhibitors (DPP4i).

COVID-19 mortality was examined in 12 studies [26, 28–38]. Seven studies with a total sample size of 19,230 people who used SGLT2 inhibitors and 217,833 people who used other glucose-lowering medications were included in the meta-analysis [28–34]. In these seven studies, one study compared users of SGLT2 inhibitors to users of glucagon-like peptide-1 receptor agonists (GLP-1RAs) and another study compared users of SGLT2 inhibitors to users of metformin. In four studies, users of SGLT2 inhibitors were compared to ‘users of other glucose lowering medications' (metformin, sulfonylureas/glinides, dipeptidyl peptidase-4 (DPP-4) inhibitors, GLP-1RAs, acarbose/pioglitazone, or insulin). The final study did not define whether nonusers of SGLT2 inhibitors used other glucose lowering medications (Table 1). SGLT2 inhibitors were associated with a 9% nonsignificant reduction in COVID-19 mortality (pooled OR 0.91; 95% CI: 0.57–1.46, *I*^2^ = 93%, random-effects model) (Figure 3). Heterogeneity between studies was high. A sensitivity analysis excluding the largest study still showed nonstatistically significant results (Supporting Information 7A) [34]. Risk of bias across the seven studies ranged from moderate to serious (Figure 2).

#### 3.3.2. COVID-19 Hospitalisation

COVID-19 hospitalisation was examined in four studies [28, 30, 37, 39]. Three studies with a total sample size of 1121 people who used SGLT2 inhibitors and 8199 people who used other glucose-lowering medications were included in the meta-analysis [28, 30, 39]. The comparator in these studies was non-SGLT2 inhibitor users including users of metformin, sulfonylureas/glinides, DPP-4 inhibitors, GLP-1RAs, acarbose/pioglitazone and insulin (Table 1). SGLT2 inhibitors were associated with a 10% nonsignificant reduction in COVID-19 hospitalisation (pooled OR 0.90; 95% CI: 0.67–1.20, *I*^2^ = 45%, random-effects model), with moderate heterogeneity between studies (Figure 3b). A sensitivity analysis excluding the largest study still showed nonsignificant results (Supporting Information 7B) [28]. There was serious risk of bias across the three studies (Figure 2).

#### 3.3.3. Pneumonia

Pneumonia was examined as an outcome in six studies [15, 16, 40–43]. Three studies with a total sample size of 217,251 people who used SGLT2 inhibitors and 229,367 people who used DPP-4 inhibitors were included in the meta-analysis [16, 40, 41]. There was a 39% reduction in pneumonia risk (pooled HR 0.61; 95% CI: 0.57–0.66, *I*^2^ = 0%, fixed-effects model) (Figure 3c). A sensitivity analysis excluding the largest study still showed statistically significant results (Supporting Information 7C) [41]. There was a moderate risk of bias across the three studies (Figure 2).

#### 3.3.4. Pneumonia-Related Mortality

Pneumonia-related mortality was examined in two studies [15, 16]. Two studies with a total sample size of 17,370 people who used SGLT2 inhibitors and 44,937 people who used DPP-4 inhibitors were included in the meta-analysis. Overall pooled estimate suggests a 51% reduction in pneumonia-related mortality (pooled HR 0.49; 95% CI: 0.35–0.67, *I*^2^ = 49%, random-effects model), with moderate heterogeneity between studies (Figure 3d). There was moderate risk of bias across the two studies (Figure 2).

#### 3.3.5. Sepsis

Sepsis was examined as an outcome in four studies [16, 43–45]. Three studies with a total sample size of 326,262 people who used SGLT2 inhibitors and 341,193 people who used other glucose-lowering medications were included in the meta-analysis. Two studies compared users of SGLT2 inhibitors to users of DPP-4 inhibitors. One study compared users of SGLT2 inhibitors to users of ‘other glucose lowering medications' (metformin, sulfonylureas, thiazolidinediones, DPP-4 inhibitors, GLP-1RAs or insulin) (Table 1). Overall pooled estimate suggests a 55% reduction in sepsis risk (pooled HR 0.45; 95% CI: 0.30–0.68, *I*^2^ = 95%, random-effects model) (Figure 3e), although heterogeneity between studies was high. A sensitivity analysis excluding the largest study still showed statistically significant results (Supporting Information 7D) [44]. There was moderate to serious risk of bias across the three studies (Figure 2).

#### 3.3.6. Other Outcomes

Several outcomes were reported exclusively by single studies. SGLT2 inhibitors were associated with reduced risk of infectious keratitis [51], infective endocarditis [50], mortality related to sepsis and infection [16, 45] and incident severe COVID-19 [46]. Conversely, SGLT2 inhibitors were not associated with Fournier's gangrene [48], hospitalisation for Fournier's gangrene [27], respiratory infection or acute upper respiratory infection [42], confirmed or suspected COVID-19 [47], severe COVID-19 outcomes [32] and COVID-19-related pneumonia or sepsis [36]. Potential reduction in hospitalisation for chronic obstructive pulmonary disease (COPD) with unspecified acute exacerbation was identified in one study [43]; however, another study found no significant association with other COPD outcomes, such as time to first moderate exacerbation or the number of moderate exacerbations [49].

### 3.4. Publication Bias

Was evaluated using funnel plot analysis (Supporting Information 8). We plotted treatment effects against study size, and the resulting symmetrical inverted funnel indicated no observed bias in the examined studies [52]. A quantitative assessment using Egger's test regression method did not show any significant results, except for sepsis (*p* = 0.0146), which indicated publication bias. Egger's test was not conducted for pneumonia-related mortality because only two studies were available.

## 4. Discussion

Our systematic review and meta-analyses of real-world evidence suggested that SGLT2 inhibitors were associated with reduced risk of pneumonia, pneumonia-related mortality, and sepsis. However, SGLT2 inhibitors were not associated with COVID-19-related hospitalisations or mortality among adults with Type 2 diabetes.

Our findings suggest SGLT2 inhibitors may reduce certain types of infection. Firstly, rates of pneumonia were lower among users of SGLT2 inhibitors than DPP-4 inhibitors. This was consistent with meta-analyses of RCTs indicating SGLT2 inhibitors may reduce pneumonia compared to placebo [53, 54]. We also observed lower pneumonia-related mortality in users of SGLT2 inhibitors than in users of DPP-4 inhibitors. The mechanisms behind the favourable effects of SGLT2 inhibitors on respiratory infections are not yet fully understood. One proposed mechanism is that SGLT2 inhibitors help reduce glucose levels in the airway surface liquid (ASL), a thin fluid layer that lines the lungs. High glucose levels in the ASL can promote bacterial growth and increase the risk of respiratory infections. By lowering glucose in this area, SGLT2 inhibitors may help protect against respiratory outcomes such as pneumonia and pneumonia-related mortality [55]. Another possibility is that the pleiotropic effects of SGLT2 inhibitors, particularly their role in weight and blood pressure control, may contribute to improved pulmonary outcomes [41]. Considering the inflammatory pathways involved in pneumonia, SGLT2 inhibitors may reduce its risk by lowering inflammation, possibly through reducing body fat [56]. Finally, we observed lower rates of sepsis among users of SGLT2 inhibitors than other glucose-lowering medications users. This was consistent with a previous meta-analysis of RCTs reporting that SGLT2 inhibitors reduced the risk of septic shock compared with placebo [53]. Sepsis and pneumonia are influenced by dysfunction in other organ systems. For example, chronic kidney disease and heart failure are independently associated with increased risk of infection and infection-related mortality among people with diabetes [57, 58]. Therefore, the known cardiorenal protective effects of SGLT2 inhibitors may indirectly contribute to the reduced risk of pneumonia and sepsis observed in our review.

We identified that treatment with SGLT2 inhibitors was not associated with COVID-19-related outcomes in patients with Type 2 diabetes. Although SGLT2 inhibitors have demonstrated cardio-renal protection and anti-inflammatory effects, these benefits may not extend to acute viral infections such as COVID-19. In our review, the COVID-19 outcomes assessed reflect more severe forms of COVID-19. Severe disease is typically driven by systemic inflammatory response syndrome, acute respiratory distress syndrome (ARDS), multiorgan dysfunction and shock [59]. These complex pathophysiological processes may limit the potential for SGLT2 inhibitors to modify outcomes once severe illness is established. This was consistent with the results of the DARE-19 trial, where dapagliflozin did not significantly reduce major clinical events among patients with cardiometabolic risk factors hospitalised with COVID-19, despite its known benefits in chronic cardiometabolic disease [60]. Our findings contrast with those of two previous reviews suggesting that SGLT2 inhibitors may protect against COVID-19-related outcomes in people with Type 2 diabetes [61, 62]. However, the authors of these reviews acknowledged that definitive conclusions were not possible due to limitations in the available data. Additionally, four systematic reviews and meta-analyses suggest that SGLT2 inhibitors were associated with reduced mortality and lower COVID-19-related adverse outcomes, whilst one review did not [63–67]. However, these meta-analyses included general population samples, patients with any type of diabetes, or only previous users of these medications.

Our study highlights several areas for future research. Further studies are needed to validate the benefit of SGLT2 inhibitors in reducing pneumonia, pneumonia-related mortality, and sepsis in patients with Type 2 diabetes and to clarify their mechanism of action. We also identified several potential outcomes for further investigation to enhance our understanding of the association between SGLT2 inhibitors and various outcomes.

Our results may have important implications for patient care and policy due to the high burden of infections in adults with Type 2 diabetes. Alongside evidence from RCTs and their meta-analyses, our findings suggest that infection risk may be a factor for clinicians and patients to consider in diabetes treatment selection, particularly for patients at high risk of infection. If the associations identified in this review are confirmed in subsequent clinical studies, clinicians might tailor prescribing of glucose-lowering medications to reduce the risk of severe infections (e.g., sepsis). Furthermore, patient education initiatives could highlight the potential benefits of SGLT2 inhibitors in reducing infection risks, potentially enhancing adherence and optimising health outcomes. Finally, with the future advent of supporting evidence, policymakers could explore revising healthcare policies to prioritise access to SGLT2 inhibitors, which may lead to a decrease in infection-related treatment rates.

Our systematic review has several strengths. To our knowledge, this is the first systematic review and meta-analysis of real-world evidence related to SGLT2 inhibitors and a comprehensive range of infection-related outcomes. Our review provides updated data on COVID-19 outcomes in Type 2 diabetes patients treated with SGLT2 inhibitors. This is important because research in the initial stages of the pandemic predominately relates to earlier SARS-CoV-2 variants. Our review utilised a comprehensive search strategy, developed with the assistance of a medical librarian, ensuring a thorough identification of relevant literature and a rigorous study selection process. We employed the validated ROBINS-I tool to independently assess the risk of bias of each observational study and reported our findings according to PRISMA and MOOSE guidelines. Additionally, we conducted a quantitative synthesis of the data, enhancing the strength and reliability of our findings.

However, several limitations should be acknowledged. Firstly, the included studies were observational, which are susceptible to possible bias. The overall risk of bias was judged to be moderate to serious, with selection bias a concern in several studies. Prevalent user designs often exclude follow-up time from treatment initiation. Prevalent users may be a select group who respond favourably to treatment and tolerate the medication well [68]. Given these findings, the conclusions drawn from this analysis should be interpreted with care, particularly for sepsis and COVID-19-related outcomes that included these studies in the meta-analysis. These results require validation through further research employing more rigorous study designs. The rigorous selection criteria applied in our study may have led to the exclusion of potentially eligible studies, as studies were excluded if authors did not specify that the population exclusively consisted of patients with Type 2 diabetes or if they did not report this as a separate analysis. Studies on COVID-19-related mortality, hospitalisation, pneumonia-related mortality and sepsis exhibited moderate to high heterogeneity, likely due to methodological differences among the included cohort studies. These studies varied in data sources, ranging from hospital or disease registries to nationwide databases. Variations in patients' clinical characteristics, SGLT2 inhibitor dosage and duration of follow-up were not assessed due to inconsistent reporting across the included studies. Notably, none of the studies reported dosage information. This inconsistency may be because our medication class or outcome of interest was not the primary focus of some studies. Evaluation of publication bias was conducted through both visual examination of funnel plots and statistical analysis. However, these findings should be interpreted with caution, as all outcomes included fewer than 10 studies, making the detection of publication bias less reliable [69, 70]. Additionally, as most studies relied on hospital administrative data (hospital discharge records or claims data) to identify infections, milder infections managed in primary care settings may have been underrepresented, with most studies capturing more severe cases requiring hospital-based care. Finally, we excluded studies in languages other than English. However, research indicates that excluding non-English language studies in systematic reviews on clinical interventions has minimal impact on the findings [71].

## 5. Conclusion

In conclusion, our systematic review and meta-analyses showed no significant association with COVID-19-related mortality or hospitalisation. However, there is an indication of reduced risk of pneumonia, pneumonia-related mortality, and sepsis. Whilst these favourable associations require future validation, they suggest potential additional benefits of SGLT2 inhibitors when prescribed for Type 2 diabetes.

## Figures and Tables

**Table 1:** Characteristics of included studies.

**First author, year Country**	**Study design**	**Study duration**	**Source of data**	**Base cohort**	**Medication regimen (type)**	**Participants (** **N** **)**	**Outcomes of interest**	**Outcome ascertainment**	**Risk estimate (95% CI)**
*COVID-19 mortality*									
[28] Hsin-Chieh, 2022 USA^a^	Retrospective cohort	2020–2021	PaTH clinical data research network	Patients with T2D and COVID-19 aged ≥ 18 years	SGLT2i (not specified)	475	ICU, intubation, death	Clinical records (ICD-10 code)	OR: 1.06 (0.73-1.55)
					Non-SGLT2i (met, DPP4i, GLP1RA)	4469			
[29] Khunti, 2022 UK^a^	Retrospective cohort	Up to Dec 2020	Nationwide audit from a network centre	Patients with T2D and hospitalised for COVID-19	SGLT2i (not specified)	228	Mortality	Clinical records (hospital audit data)	OR: 1.13 (0.78-1.63)
					Non-SGLT2i (not specified)	2811			
[30] Mannucci, 2022 Italy^a^	Retrospective cohort	1 Mar 2020–31 Dec 2020	COVID-19 surveillance registry	Patients with T2D and COVID-19 aged ≥ 18 years	SGLT2i (dapagliflozin, canagliflozin, empagliflozin, ertugliflozin, ipragliflozin, luseogliflozin, tofogliflozin, sotagliflozin, canagliflozin-met, dapagliflozin-met, empagliflozin-met, ertugliflozin-met, dapagliflozin-saxagliptin, ertugliflozin-sitagliptin)	557	Mortality	Claims database + surveillance registry (ICD-9 code)	OR: 1.28 (0.65-2.53)
					Non-SGLT2i (Insulin, met, SU/glinides, acarbose/pioglitazone, DPP4i, GLP1RA)	3223			
[31] Monda, 2023 Italy^a^	Retrospective cohort	Jan 2020–Dec 2021	Hospitalisation data	Patients with T2D hospitalised for COVID-19 aged > 18 years	SGLT2i (dapagliflozin, canagliflozin, empagliflozin, ertugliflozin, ipragliflozin, luseogliflozin, tofogliflozin, sotagliflozin)	326	In-hospital death	Hospital discharge data (ICD-9/10 codes)	OR: 1.42 (0.6-2.1)
					GLP1RA (exenatide, liraglutide, lixisenatide, albiglutide, dulaglutide, semaglutide, efpeglenatide)	326			
[32] Ouchi, 2022 Spain^a^	Retrospective cohort	Mar 2020–Jun 2020	SIDIAP	Adult patients withT2D and COVID-19 with records in PHC	SGLT2i (not specified)	1809	Mortality	Clinical records (primary care EHR, ICD-10 code)	OR: 0.69 (0.25-1.93)
					Met	6168			
[33] Ramos-Rincón, 2021 Spain^a^	Retrospective cohort	1 Mar 2020–29 May 2020	SEMICOVID-19 Registry	Patients with T2D hospitalised with COVID-19 aged ≥ 80 years	SGLT2i (not specified)	32	In-hospital death	Registry data	OR: 0.81 (0.75-1.99)
					Non-SGLT2i (met, DPP4i, insulin, GLP1RA)	758			
[34] Shestakova, 2022 Russia^a^	Retrospective cohort	20 Mar 2020–25 Nov 2021	NDR	Patients with T2D and COVID-19	SGLT2i (not specified)	17092	Case fatality rate	Registry data	**OR: 0.46 (0.44-0.49)**
					Non-SGLT2i (Insulin, met, SU, DPP4i, GLP1RA)	207098			
[26] Araldi, 2023 UK (preprint)	Prospective cohort	2006–2010–20 Mar 2021	UK Biobank	Patients with T2D aged 38-73 years	SGLT2i (not specified)	1821	Mortality	Registry data (ICD-10 code)	0.44% v. 0.37% p value 0.58
					Non-SGLT2i (Met, SU, DPP4i, insulin, GLP1RA, TZD)	1821			
[35] Khunti, 2021 UK	Retrospective cohort	16 Feb 2020–31 Aug 2020	NDA	Patients with T2D and registered with general practice	SGLT2i (not specified)	266505	Mortality	Hospital discharge data (ICD-10 code)	**HR: 0.82 (0.74-0.91)**
					Non-SGLT2i (DPP4i, GLP1RA, insulin, meglitinides, met, SU, TZD, and AGIs)	2584960			
[36] Lim, 2024 South Korea	Retrospective cohort	Jan 2018–Apr 2022	HIRA	Patients with T2D and COVID-19	SGLT2i (not specified)	40776	All-cause mortality	Claims database (ICD-10 code)	HR: 0.80 (0.64-1.01)
					Non-SGLT2i (not specified)	40776			
[37] Min, 2022 USA	Retrospective cohort	15 Mar 2020–15 Jun 2020	INSIGHT	Adult patients with T2D hospitalised with COVID-19	SGLT2i (dapagliflozin, canagliflozin, empagliflozin, ertugliflozin, ipragliflozin, luseogliflozin, tofogliflozin, sotagliflozin, ertugliflozin-met, dapagliflozin-saxagliptin, empagliflozin-linagliptin, ertugliflozin-sitagliptin, ertugliflozin-linagliptin, dapagliflozin-linagliptin) + Met	8248	In-hospital death	Clinical records (ICD-10 code)	HR: 1.18 (0.58-2.43)
					Non-SGLT2i (SU, DPP4i, GLP1RA) + Met	22499			
[38] Pérez-Belmonte, 2020 Spain	Retrospective cohort	1 Mar 2020–19 Jul 2020	SEMI-COVID-19 Registry	Patients with T2D hospitalised with COVID-19	SGLT2i (not specified) + Met	34	In-hospital death	Registry data	**19.4% vs 39.1% p-value 0.003**
					Non-SGLT2i (met, DPP4i, insulin, met + DPP4i, met + insulin)	34			

COVID-19 hospitalisation									
[28] Hsin-Chieh, 2022 USA^a^	Retrospective cohort	2020–2021	PaTH clinical data research network	Patients with T2D and COVID-19 aged ≥ 18 years	SGLT2i (not specified)	475	Hospitalisation	Clinical records (ICD-10 code)	OR: 1.08 (0.82-1.42)
					Non-SGLT2i (met, DPP4i, GLP1RA)	4469			
[30] Mannucci, 2022 Italy^a^	Retrospective cohort	1 Mar 2020–31 Dec 2020	COVID-19 surveillance registry	Patients with T2D and COVID-19 aged ≥ 18 years	SGLT2i (dapagliflozin, canagliflozin, empagliflozin, ertugliflozin, ipragliflozin, luseogliflozin, tofogliflozin, sotagliflozin, canagliflozin and met, dapagliflozin and met, empagliflozin and met, ertugliflozin and met, dapagliflozin and saxagliptin, ertugliflozin and sitagliptin)	557	Hospitalisation	Claims database + surveillance registry (ICD-9 code)	OR: 0.89 (0.62-1.29)
					Non-SGLT2i (Insulin, met, SU/glinides, acarbose/pioglitazone, DPP4i, GLP1RA)	3223			
[39] Piarulli, 2023 Italy^a^	Retrospective cohort	Feb 2020–Feb 2021	Claim databases, hospital discharge records, and clinical records	Patients with T2D and COVID-19 in a local health district of Veneto	SGLT2i (not specified)	190	Hospitalisation	Claim databases, hospital discharge records, and clinical records	OR: 0.61 (0.36-1.04)
					Non-SGLT2i (Insulin, DPP4i, GLP1RA, met, pioglitazone, SU)	2263			
[37] Min, 2022 USA	Retrospective cohort	15 Mar 2020–15 Jun 2020	INSIGHT	Adult patients with T2D hospitalised with COVID-19	SGLT2i (dapagliflozin, canagliflozin, empagliflozin, ertugliflozin, ipragliflozin, luseogliflozin, tofogliflozin, sotagliflozin, ertugliflozin-met, dapagliflozin-saxagliptin, empagliflozin-linagliptin, ertugliflozin-sitagliptin, ertugliflozin-linagliptin, dapagliflozin-linagliptin) + Met	8248	Hospitalisation	Clinical records (ICD-10 code)	HR: 1.09 (0.78-1.53)
					Non-SGLT2i (SU, DPP4i, GLP1RA) + Met	22499			

Pneumonia									
[40] Brunetti, 2021 UK^a^	Retrospective cohort	2013–2017	CPRD	Patients with T2D aged ≥ 18 years	SGLT2i (dapagliflozin, canagliflozin, empagliflozin)	1011	Hospitalisation for HCAP	Hospital discharge data (ICD-10 code)	**HR: 0.48 (0.28-0.82)**
					DPP4i (not specified)	5552			
[41] Jeong, 2023 South Korea^a^	Retrospective cohort	Jan 2016–Dec 2020	HIRA	Patients with T2D aged ≥ 18 years	SGLT2i (dapagliflozin, empagliflozin, ertugliflozin, ipragliflozin)	205534	Pneumonia	Claims database (ICD-10 code)	**HR: 0.61 (0.56-0.66)**
					DPP4i (sitagliptin, vildagliptin, saxagliptin, alogliptin, linagliptin, teneligliptin, anagliptin, trelagliptin)	205534			
[16] Wu, 2022 Hong Kong^a^	Retrospective cohort	1 Jan 2015–31 Jan 2021	CDARS	Hospitalised patients with T2D aged ≥18 years	SGLT2i (canagliflozin, dapagliflozin, or empagliflozin)	10706	Pneumonia	Hospital discharge data (ICD-9 code)	**HR 0.63 (0.55-0.72)**
					DPP4i (alogliptin, linagliptin, linagliptin-met, saxagliptin, sitagliptin, sitagliptin-met, vildagliptin, or vildagliptin-met)	18281			
[15] Au, 2022 Hong Kong	Retrospective cohort	2015–2018	CDARS	Patients with T2D aged ≥ 30 years	SGLT2i (canagliflozin, dapagliflozin, empagliflozin, ertugliflozin)	6664	Pneumonia	Hospital discharge data (ICD-9/10 codes)	**IRR: 0.71 (0.62-0.81)**
					DPP4i (sitagliptin, vildagliptin, saxagliptin, linagliptin, alogliptin, omarigliptin)	26656			
[42] Park, 2023 South Korea	Retrospective cohort	1 Jan 2019–31 Dec 2019	HIRA	Patients aged ≥ 18 years with T2D with hospital encounters	SGLT2i (not specified) + insulin	306	Influenza or pneumonia	Claims database (ICD-10 code)	OR 1.51 (0.78-2.94)
					Met + insulin	587			
[43] Tan, 2024 Australia	Retrospective cohort	1 Dec 2013–30 June 2019	NDSS	Patients with T2D who resided in Victoria, New South Wales and Queensland	SGLT2i (dapagliflozin, empagliflozin, ertugliflozin, canagliflozin, dapagliflozin + met, empagliflozin + met, ertugliflozin + met)	99569	Unspecified pneumonia	Hospital discharge data (ICD-10-AM code)	**IRR: 0.64 (0.56-0.72)**
					DPP-4i (sitagliptin, vildagliptin, saxagliptin, alogliptin, linagliptin, sitagliptin + met, vildagliptin + met, saxagliptin + met, linagliptin + met, alogliptin + met, sitagliptin + simvastatin)	186353			

Pneumonia-related mortality									
[15] Au, 2022 Hong Kong^a^	Retrospective cohort	2015–2018	CDARS	Patients with T2D aged ≥ 30 years	SGLT2i (canagliflozin, dapagliflozin, empagliflozin, ertugliflozin)	6664	Pneumonia-mortality	Hospital discharge data (ICD-9/10 codes)	**HR: 0.57 (0.42-0.77)**
					DPP4i (sitagliptin, vildagliptin, saxagliptin, linagliptin, alogliptin, omarigliptin)	26656			
[16] Wu, 2022 Hong Kong^a^	Retrospective cohort	1 Jan 2015–31 Jan 2021	CDARS	Hospitalised patients with T2D aged ≥ 18 years	SGLT2i (canagliflozin, dapagliflozin, or empagliflozin)	10706	Pneumonia- death	Hospital discharge data (ICD-9 code)	**HR 0.41 (0.29-0.58)**
					DPP4i (alogliptin, linagliptin, linagliptin-met, saxagliptin, sitagliptin, sitagliptin-met, vildagliptin, or vildagliptin-met)	18281			

Sepsis									
[44] Hu, 2024 Taiwan^a^	Retrospective cohort	2016–2019	NHIRD	Patients with T2D > 20 years	SGLT2i (dapagliflozin, canagliflozin, empagliflozin, ertugliflozin, ipragliflozin, luseogliflozin, tofogliflozin, sotagliflozin)	305248	Risk of sepsis/septic shock	Claims database (ICD-9/10 codes)	**HR: 0.63 (0.59-0.66)**
					Non-SGLT2i (met, insulin, DPP4i, GLP1RA, TZD, SU)	305248			
[45] Ng, 2023 Hong Kong^a^	Retrospective cohort	1 Jan 2015–31 Dec 2019	CDARS	Patients with T2D aged ≥18 years	SGLT2i (canagliflozin, dapagliflozin, empagliflozin)	10308	ICU admissions for sepsis	Hospital discharge data (ICD-9 code)	**HR: 0.61 (0.43-0.85)**
					DPP4i (alogliptin, linagliptin, linagliptin/met, saxagliptin, sitagliptin, sitagliptin/met, vildagliptin, and vildagliptin/met)	17664			
[16] Wu, 2022 Hong Kong^a^	Retrospective cohort	1 Jan 2015–31 Jan 2021	CDARS	Hospitalised patients with T2D aged ≥ 18 years	SGLT2i (canagliflozin, dapagliflozin, or empagliflozin)	10706	Sepsis	Hospital discharge data (ICD-9 code)	**HR 0.52 (0.44-0.62)**
					DPP4i (alogliptin, linagliptin, linagliptin-met, saxagliptin, sitagliptin, sitagliptin-met, vildagliptin, or vildagliptin-met)	18281			
[43] Tan, 2024 Australia	Retrospective cohort	1 Dec 2013–30 June 2019	NDSS	Patients with T2D who resided in Victoria, New South Wales and Queensland	SGLT2i (dapagliflozin, empagliflozin, ertugliflozin, canagliflozin, dapagliflozin + met, empagliflozin + met, ertugliflozin + met)	99569	Unspecified sepsis	Hospital discharge data (ICD-10-AM code)	**IRR: 0.60 (0.51-0.72)**
					DPP-4i (sitagliptin, vildagliptin, saxagliptin, alogliptin, linagliptin, sitagliptin + met, vildagliptin + met, saxagliptin + met, linagliptin + met, alogliptin + met, sitagliptin + simvastatin)	186353			

Other outcomes									
[32] Ouchi, 2022 Spain	Retrospective cohort	Mar 2020–30 Jun 2020	SIDIAP	Adult patients with T2D and COVID-19 with records in PHC	SGLT2i (not specified)	1809	Severe COVID-19 outcomes	Clinical records (ICD-10 code)	OR: 0.89 (0.52-1.53)
					Met	6168			
[46] Ferrannini, 2022 Sweden	Retrospective cohort	1 Feb 2020–15 May 2021	NPR	Patients with T2D and COVID-19 aged ≥ 18 years	SGLT2i (dapagliflozin, canagliflozin, empagliflozin, ertugliflozin, ipragliflozin, luseogliflozin, tofogliflozin, sotagliflozin, dapagliflozin and met, empagliflozin-met, ertugliflozin-met, dapagliflozin-saxagliptin, empagliflozin-linagliptin, ertugliflozin-sitagliptin, ertugliflozin-linagliptin, dapagliflozin-linagliptin)	39172	Incident severe COVID-19	Registry data (ICD-10 code)	**RR 1.11 (1.02-1.22)**
					Non-SGLT2i (DPP4i GLP1RA, insulin, met, another oral antidiabetic)	305241			
[36] Lim, 2024 South Korea	Retrospective cohort	Jan 2018–Apr 2022	HIRA	Patients with T2D and COVID-19	SGLT2i (not specified)	40776	COVID-19 Pneumonia	Claims database (ICD-10)	HR: 1.03 (0.91-1.16)
					Non-SGLT2i (not specified)	40776	COVID-19 Sepsis		HR: 0.78 (0.46-1.31)
[47] Sainsbury, 2021 UK	Retrospective cohort	30 Jan 2020–22 Jul 2020	THIN	Patients with T2D aged ≥ 18 years	SGLT2i (not specified)	7676	Confirmed or suspected COVID-19	Primary care records	HR 0.92 (0.66-1.29)
					DPP4i (not specified)	7676	Confirmed COVID-19		HR 0.78 (0.39-1.56)
[45] Ng, 2023 Hong Kong^a^	Retrospective cohort	1 Jan 2015–31 Dec 2019	CDARS	Patients with T2D aged ≥ 18 years	SGLT2i (canagliflozin, dapagliflozin, empagliflozin)	10308	Mortality due to Infections	Hospital discharge data (ICD-9 code)	**HR: 0.26 (0.2-0.34)**
					DPP4i (alogliptin, linagliptin, linagliptin/met, saxagliptin, sitagliptin, sitagliptin/met, vildagliptin, and vildagliptin/met)	17664			
[16] Wu, 2022 Hong Kong^a^	Retrospective cohort	1 Jan 2015–31 Jan 2021	CDARS	Hospitalised patients with T2D aged ≥ 18 years	SGLT2i (canagliflozin, dapagliflozin, or empagliflozin)	10706	Sepsis-death	Hospital discharge data (ICD-9 code)	**HR 0.39 (0.18-0.84)**
					DPP4i (alogliptin, linagliptin, linagliptin-met, saxagliptin, sitagliptin, sitagliptin-met, vildagliptin, or vildagliptin-met)	18281			
[42] Park, 2023 South Korea	Retrospective cohort	1 Jan 2019–31 Dec 2019	HIRA	Patients aged ≥ 18 years with T2D with hospital encounters	SGLT2i (not specified) + insulin	306	Respiratory infection	Claims database (ICD-10 code)	OR 0.89 (0.67-1.2)
					Met + insulin	587	Acute upper respiratory infection		OR 0.87 (0.65-1.17)
[48] Petruski-Ivleva, 2020 USA	Retrospective cohort	2014–2017	Administrative data from Horizon Blue Cross Blue Shield of New Jersey	Patients with T2D receiving second line antihyperglycemics	SGLT2i (not specified)	3493	FG or necrotizing fasciitis	Hospital discharge data (ICD-9/10 codes)	RR: 1.81 (0.16-19.96)
					DPP4i (not specified)	3493			
					SGLT2i (not specified)	2212	FG or necrotizing fasciitis		RR: 0.47 (0.04-5.13)
					GLP1RA (not specified)	2212			
					SGLT2i (not specified)	2731	FG or necrotizing fasciitis		RR: 0.96 (0.06-15.32)
					SU (not specified)	2731			
[27] Wang, 2020 USA	NCC within CS (retrospective)	1 Apr 2013–31 Mar 2018	US Truven MarketScan	Patients with T2D aged ≥ 18 years	SGLT2i (not specified)	109	Hospitalisation for FG	Administrative data (ICD-9/10 codes)	OR: 0.55 (0.25-1.18)
					Non-SGLT2i (Met, SU, TZD, acarbose, meglitinides, DPP4i, GLP1RA or insulin)	571			
[49] Pradhan, 2022 UK	Retrospective cohort	1 Jan 2013–30 Dec 2019–31 Mar 2020	GOLD and CPRD	Patients with T2D and COPD (who received coprescription of a corticosteroid and antibiotic)	SGLT2i (canagliflozin, dapagliflozin, empagliflozin)	2956	Time to first COPD exacerbation: Moderate	Hospital discharge data (ICD-10 code)	HR: 1.02 (0.83-1.27)
					SU (glibenclamide, gliclazide, glipizide, glimepiride, tolbutamide)	10841	Number of COPD exacerbations: Moderate		RR: 1.00 (0.79-1.26)
[43] Tan, 2024 Australia	Retrospective cohort	1 Dec 2013–30 June 2019	NDSS	Patients with T2D who resided in Victoria, New South Wales and Queensland	SGLT2i (dapagliflozin, empagliflozin, ertugliflozin, canagliflozin, dapagliflozin + met, empagliflozin + met, ertugliflozin + met)	99569	Hospitalisation for COPD with unspecified acute exacerbation	Hospital discharge data (ICD-10-AM code)	**IRR: 0.66 (0.57-0.78)**
					DPP-4i (sitagliptin, vildagliptin, saxagliptin, alogliptin, linagliptin, sitagliptin + met, vildagliptin + met, saxagliptin + met, linagliptin + met, alogliptin + met, sitagliptin + simvastatin)	186353			
[50] Chou, 2023 (Preprint) Hong-Kong	Retrospective cohort	1 Jan 2015–31 Dec 2020	CDARS	Patients with T2D ≥ 18 years	SGLT2i (not specified)	28774	New-onset infective endocarditis	Hospital discharge data (ICD-9/10 codes)	**HR: 0.58 (0.41-0.81)**
					DPP4i (not specified)	28774			
[51] Tsai, 2024 Taiwan	Retrospective cohort	1 Jan 2014–31 Dec 2020	NHIRD	Patients with T2D aged 20–100 years	SGLT2i (empagliflozin, dapagliflozin, canagliflozin, ertugliflozin)	41724	Infectious keratitis	Claims database (ICD-9/10 codes)	**HR: 0.654 (0.537-0.796)**
					Non-SGLT2i (biguanides, SU, AGIs, TZD, DPP4i, and insulin)	41724			

*Note:* Bold: significant results.

Abbreviations: AGIs (Alpha-glucosidase inhibitors); CAP (Community-acquired pneumonia); CDARS (Clinical Data Analysis and Reporting System); COPD (Chronic obstructive pulmonary disease); COVID-19 (Coronavirus disease); CPRD (Clinical Practice Research Datalink); EHR (Electronic Health Records); GLD (Glucose-lowering Drug); GLP1RA (Glucagon-like peptide-1 agonists); GOLD (Gp OnLine Data); FG (Fournier's gangrene); HR (Hazard Ratio); HCAP (Hospitalization for community-acquired pneumonia); HbA1c (Glycated haemoglobin); HIRA (Health Insurance Review and Assessment Service database of South Korea); ICD-9 (International Classification of Diseases, Ninth Revision); ICD-10 (International Classification of Diseases, Tenth Revision; ICD-10-AM (International Classification of Diseases, Tenth Revision, Australian Modification); ICU (Intensive care unit); IRR (Incidence Rate Ratio); Met (Metformin); NDA (National Diabetes Audit); NDR (National Diabetes Register); NDSS (National Diabetes Services Scheme); NHIRD (Taiwan's National Health Insurance Research Database); NCC (Nested-case control); NPR (National Patient Registry); OR (Odds Ratio); PaTH (Toward a Learning Health System clinical data research network); PHC (Primary Health Care); SGLT2i (Sodium-glucose cotransporter 2 inhibitors); SEMI-COVID-19 (Spanish Society of Internal Medicine's registry of COVID-19 patients registry); SIDIAP (Information System for Research in Primary Care); SU (Sulfonylureas); THIN (The Health Improvement Network); TZD (Thiazolidinediones); T2D (Type 2 Diabetes); UK (United Kingdom); USA (United States of America).

^a^Included in meta-analysis.

## Data Availability

Data were extracted from publicly available research papers. All datasets generated during this study can be accessed upon reasonable request from the corresponding author.

## Supporting Information

Supporting Information Supporting Information 1: Search strategy. Supporting Information 2: PRISMA 2020 checklist. Supporting Information 3: MOOSE (Meta-analyses Of Observational Studies in Epidemiology) Checklist. Supporting Information 4: Full text exclusions. Supporting Information 5: Additional details for each individual study included. Supporting Information 6: Risk of bias assessments. Supporting Information 7: Sensitivity analyses. Supporting Information 8: Funnel plots to judge the publication bias.
