# Supplementary material for: SGLT2 Inhibitors and the Risk of Infections in Type 2 Diabetes: Systematic Review and Meta-Analyses of Real-World Evidence
Source: J Diabetes Res. 2025 Oct 16;2025:5888495. doi: 10.1155/jdr/5888495 (PMC12549203; doi:10.1155/jdr/5888495)
Supplement: Supporting Information — 1: Search strategy. Supporting Information 2: PRISMA 2020 checklist. Supporting Information 3: MOOSE (Meta-analyses Of Observational Studies in Epidemiology) Checklist. Supporting Information 4: Full text exclusions. Supporting Information 5: Additional details for each individual study included. Supporting Information 6: Risk of bias assessments. Supporting Information 7: Sensitivity analyses. Supporting Information 8: Funnel plots to judge the publication bias. [file 5888495.f1.docx]

**SGLT2 inhibitors and the risk of infections in type 2 diabetes: systematic review and meta-analyses of real-world evidence**

Maria Jose Alfonso Arvez^1^, George SQ Tan^1^, Miriam T. Y. Leung^1^, Zanfina Ademi^1^, PhD, Simon Bell, PhD^1^

1. Monash Institute of Pharmaceutical Sciences Centre for Medicine Use and Safety, Parkville, Australia

Contents

[Supplementary 1 – Search strategy 2](#_Toc201591987)

[Ovid MEDLINE 2](#_Toc201591988)

[Embase 2](#_Toc201591989)

[Scopus 3](#_Toc201591990)

[Google scholar 3](#_Toc201591991)

[Supplementary 2 – PRISMA 2020 checklist 4](#_Toc201591992)

[Supplementary 3 – MOOSE (Meta-analyses Of Observational Studies in Epidemiology) Checklist 7](#_Toc201591993)

[Supplementary 4 – Full text exclusions 9](#_Toc201591994)

[Supplementary 5 – Additional details for each individual study included 15](#_Toc201591995)

[Supplementary 6 – Risk of bias assessments 19](#_Toc201591996)

[Supplementary 7 – Sensitivity analyses 41](#_Toc201591997)

[Supplementary 8 – Funnel plots to judge the publication bias 43](#_Toc201591998)

# Supplementary 1 – Search strategy

## Ovid MEDLINE

1 exp Sodium-Glucose Transporter 2 Inhibitors/ 6313

2 sodium glucose.ti,ab. 6983

3 sglt*.ti,ab. 7472

4 1 or 2 or 310547

5 exp Observational Studies as Topic/ 9135

6 observational.ti,ab. 222736

7 clinical practice.ti,ab. 214263

8 real world.ti,ab. 57722

9 exp Cohort Studies/ 2536116

10 cohort study.ti,ab. 251452

11 case control.ti,ab. 137087

125 or 6 or 7 or 8 or 9 or 10 or 11 2976081

13 effect*.ti,ab. 7140659

14 exp patient safety/ 25653

15 safety.ti,ab. 576788

16 exp "Drug-Related Side Effects and Adverse Reactions"/ 133577

17 (risk adj3 events).ti,ab. 22578

18 infection*.ti,ab. 1503412

19 outcome*.ti,ab. 2007883

20 13 or 14 or 15 or 16 or 17 or 18 or 19 9760266

21 4 and 12 and 20 1321

22 limit 21 to (english language and humans and yr="2012 -Current") 1262

## Embase Classic+Embase <1947 to 2023 November 03>

1 exp Sodium-Glucose Transporter 2 Inhibitors/ 26525

2 sodium glucose.ti, ab.12846

3 sglt*.ti,ab. 16028

4 1 or 2 or 331446

5 exp Observational Studies as Topic/ 342638

6 observational.ti,ab. 425566

7 clinical practice.ti,ab .375173

8 real world.ti,ab. 137101

9 exp Cohort Studies/ 1066404

10 cohort study.ti,ab. 429791

11 case control.ti,ab. 207181

12 5 or 6 or 7 or 8 or 9 or 10 or 11 2147335

13 effect*.ti,ab. 10956204

14 exp patient safety/ 167679

15 safety.ti,ab. 1074840

16 exp "Drug-Related Side Effects and Adverse Reactions"/ 659595

17 (risk adj3 events).ti,ab. 42777

18 infection*.ti,ab. 2348205

19 outcome*.ti,ab. 3522271

20 13 or 14 or 15 or 16 or 17 or 18 or 19 15516431

21 4 and 12 and 20 3867

22 limit 21 to (english language and humans and yr="2012 -Current") 3750

## Scopus

TITLE-ABS ( ( “sodium glucose” OR "transporter 2 inhibitor*" OR sglt* ) AND ( "observational studies" OR observational OR "clinical practice" OR "real world" OR "cohort stud*" OR "case control" ) AND ( effect* OR "patient safety" OR safety OR "drug related side effects" OR "adverse reactions" OR infection* OR outcome* ) ) AND PUBYEAR > 2011 AND PUBYEAR < 2024 AND ( LIMIT-TO ( LANGUAGE , "English" ) ) AND ( LIMIT-TO ( DOCTYPE , "ar" ) ) AND ( LIMIT-TO ( EXACTKEYWORD , "Human" ) )

## Google scholar

("sodium glucose" OR "transporter 2 inhibitor*" OR sglt*) AND ("observational studies" OR observational OR "clinical practice" OR "real world" OR "cohort stud*" OR "case control") AND (effect* OR "patient safety" OR safety OR "drug related side effects" OR "adverse reactions" OR infection* OR outcome*) after:2011

| Supplementary 2 – PRISMA 2020 checklist | | | |
| --- | --- | --- | --- |
| Section and Topic | Item # | Checklist item | Location where item is reported |
| TITLE | | |  |
| Title | 1 | Identify the report as a systematic review. | Page 2 |
| ABSTRACT | | |  |
| Abstract | 2 | See the PRISMA 2020 for Abstracts checklist. | Page 2, 3 |
| INTRODUCTION | | |  |
| Rationale | 3 | Describe the rationale for the review in the context of existing knowledge. | Page 3, 4 |
| Objectives | 4 | Provide an explicit statement of the objective(s) or question(s) the review addresses. | Page 4 |
| METHODS | | |  |
| Eligibility criteria | 5 | Specify the inclusion and exclusion criteria for the review and how studies were grouped for the syntheses. | Page 5, 6 |
| Information sources | 6 | Specify all databases, registers, websites, organisations, reference lists and other sources searched or consulted to identify studies. Specify the date when each source was last searched or consulted. | Page 5 |
| Search strategy | 7 | Present the full search strategies for all databases, registers and websites, including any filters and limits used. | Supplementary 1 |
| Selection process | 8 | Specify the methods used to decide whether a study met the inclusion criteria of the review, including how many reviewers screened each record and each report retrieved, whether they worked independently, and if applicable, details of automation tools used in the process. | Page 6 |
| Data collection process | 9 | Specify the methods used to collect data from reports, including how many reviewers collected data from each report, whether they worked independently, any processes for obtaining or confirming data from study investigators, and if applicable, details of automation tools used in the process. | Page 6 |
| Data items | 10a | List and define all outcomes for which data were sought. Specify whether all results that were compatible with each outcome domain in each study were sought (e.g. for all measures, time points, analyses), and if not, the methods used to decide which results to collect. | Page 6 |
|  | 10b | List and define all other variables for which data were sought (e.g. participant and intervention characteristics, funding sources). Describe any assumptions made about any missing or unclear information. | Page 6 |
| Study risk of bias assessment | 11 | Specify the methods used to assess risk of bias in the included studies, including details of the tool(s) used, how many reviewers assessed each study and whether they worked independently, and if applicable, details of automation tools used in the process. | Page 7 |
| Effect measures | 12 | Specify for each outcome the effect measure(s) (e.g. risk ratio, mean difference) used in the synthesis or presentation of results. | Page 7, 8 |
| Synthesis methods | 13a | Describe the processes used to decide which studies were eligible for each synthesis (e.g. tabulating the study intervention characteristics and comparing against the planned groups for each synthesis (item #5)). | Page 6, 7 |
|  | 13b | Describe any methods required to prepare the data for presentation or synthesis, such as handling of missing summary statistics, or data conversions. | Page 6, 7 |
|  | 13c | Describe any methods used to tabulate or visually display results of individual studies and syntheses. | Page 6, 7 |
|  | 13d | Describe any methods used to synthesize results and provide a rationale for the choice(s). If meta-analysis was performed, describe the model(s), method(s) to identify the presence and extent of statistical heterogeneity, and software package(s) used. | Page 6, 7 |
|  | 13e | Describe any methods used to explore possible causes of heterogeneity among study results (e.g. subgroup analysis, meta-regression). | Page 7 |
|  | 13f | Describe any sensitivity analyses conducted to assess robustness of the synthesized results. | Page 7 |
| Reporting bias assessment | 14 | Describe any methods used to assess risk of bias due to missing results in a synthesis (arising from reporting biases). | Page 7, 8 |
| Certainty assessment | 15 | Describe any methods used to assess certainty (or confidence) in the body of evidence for an outcome. | Page 7 |
| RESULTS | | |  |
| Study selection | 16a | Describe the results of the search and selection process, from the number of records identified in the search to the number of studies included in the review, ideally using a flow diagram. | Page 8, 9, Figure 1 |
|  | 16b | Cite studies that might appear to meet the inclusion criteria, but which were excluded, and explain why they were excluded. | Page 8, 9, Supplementary 4 |
| Study characteristics | 17 | Cite each included study and present its characteristics. | Page 8, 9, Table 1 |
| Risk of bias in studies | 18 | Present assessments of risk of bias for each included study. | Figure 3, Supplementary 6 |
| Results of individual studies | 19 | For all outcomes, present, for each study: (a) summary statistics for each group (where appropriate) and (b) an effect estimates and its precision (e.g. confidence/credible interval), ideally using structured tables or plots. | Page 9-12, Supplementary 5 |
| Results of syntheses | 20a | For each synthesis, briefly summarise the characteristics and risk of bias among contributing studies. | Page 9-12, Supplementary 6 |
|  | 20b | Present results of all statistical syntheses conducted. If meta-analysis was done, present for each the summary estimate and its precision (e.g. confidence/credible interval) and measures of statistical heterogeneity. If comparing groups, describe the direction of the effect. | Page 9-12 |
|  | 20c | Present results of all investigations of possible causes of heterogeneity among study results. | Page 9-12 |
|  | 20d | Present results of all sensitivity analyses conducted to assess the robustness of the synthesized results. | Page 9-12, Supplementary 7 |
| Reporting biases | 21 | Present assessments of risk of bias due to missing results (arising from reporting biases) for each synthesis assessed. | Page 12, 13, Supplementary 8 |
| Certainty of evidence | 22 | Present assessments of certainty (or confidence) in the body of evidence for each outcome assessed. | Page 9-12 |
| DISCUSSION | | |  |
| Discussion | 23a | Provide a general interpretation of the results in the context of other evidence. | Page 13 |
|  | 23b | Discuss any limitations of the evidence included in the review. | Page 15-16 |
|  | 23c | Discuss any limitations of the review processes used. | Page 15-16 |
|  | 23d | Discuss implications of the results for practice, policy, and future research. | Page 14 |
| OTHER INFORMATION | | |  |
| Registration and protocol | 24a | Provide registration information for the review, including register name and registration number, or state that the review was not registered. | Page 2, 4 |
|  | 24b | Indicate where the review protocol can be accessed, or state that a protocol was not prepared. | Page 4 |
|  | 24c | Describe and explain any amendments to information provided at registration or in the protocol. | NA |
| Support | 25 | Describe sources of financial or non-financial support for the review, and the role of the funders or sponsors in the review. | Page 17, 18 |
| Competing interests | 26 | Declare any competing interests of review authors. | Page 17 |
| Availability of data, code and other materials | 27 | Report which of the following are publicly available and where they can be found: template data collection forms; data extracted from included studies; data used for all analyses; analytic code; any other materials used in the review. | Page 18 |

# Supplementary 3 – MOOSE (Meta-analyses Of Observational Studies in Epidemiology) Checklist

| Reporting Criteria | Reported (Yes/No) | Reported on Page No. |
| --- | --- | --- |
| Reporting of Background | | |
| Problem definition | Yes | 3, 4 |
| Hypothesis statement | Yes | 3, 4 |
| Description of Study Outcome(s) | Yes | 3, 4 |
| Type of exposure or intervention used | Yes | 3, 4 |
| Type of study design used | Yes | 3, 4 |
| Study population | Yes | 3, 4 |
| Reporting of Search Strategy | | |
| Qualifications of searchers (e.g., librarians and investigators) | Yes | 5, 6 |
| Search strategy, including time period included in the synthesis and keywords | Yes | 5, Suppl. 1 |
| Effort to include all available studies, including contact with authors | Yes | 5 |
| Databases and registries searched | Yes | 5 |
| Search software used, name and version, including special features used (e.g., explosion) | Yes | 6, Suppl. 1 |
| Use of hand searching (e.g., reference lists of obtained articles) | Yes | 6-7 |
| List of citations located and those excluded, including justification | Yes | Figure 1, Suppl. 4 |
| Method for addressing articles published in languages other than English | Yes | 5 |
| Method of handling abstracts and unpublished studies | Yes | 5 |
| Description of any contact with authors | Yes | 5 |
| Reporting of Methods | | |
| Description of relevance or appropriateness of studies assembled for assessing the hypothesis to be tested | Yes | 5 |
| Rationale for the selection and coding of data (e.g., sound clinical principles or convenience) | Yes | 5 |
| Documentation of how data were classified and coded (e.g., multiple rates, blinding, and interrater reliability) | Yes | 5 |
| Assessment of confounding (e.g., comparability of cases and controls in studies where appropriate) | Yes | 7 |
| Assessment of study quality, including blinding of quality assessors; stratification or regression on possible predictors of study results | Yes | 7 |
| Assessment of heterogeneity | Yes | 6 |
| Description of statistical methods (e.g., complete description of fixed or random effects models, justification of whether the chosen models account for predictors of study results, dose-response models, or cumulative meta-analysis) in sufficient detail to be replicated | Yes | 8-9 |
| Provision of appropriate tables and graphics | Yes | Table 1, Figures 1-3 |
| Reporting of Results |  |  |
| Table giving descriptive information for each study included | Yes | Table 1, Suppl. 5 |
| Results of sensitivity testing (e.g., subgroup analysis) | Yes | 9-12, Suppl. 7 |
| Indication of statistical uncertainty of findings | Yes | 9-12 |
| Reporting of Discussion |  |  |
| Quantitative assessment of bias (e.g., publication bias) | Yes | 12, 13, Suppl. 8 |
| Justification for exclusion (e.g., exclusion of non–English-language citations) | Yes | 16 |
| Assessment of quality of included studies | Yes | 9, Suppl. 6 |
| Reporting of Conclusions |  |  |
| Consideration of alternative explanations for observed results | Yes | 13-16 |
| Generalization of the conclusions (i.e., appropriate for the data presented and within the domain of the literature review) | Yes | 16 |
| Guidelines for future research | Yes | 16 |
| Disclosure of funding source | Yes | 17 |

# Supplementary 4 – Full text exclusions

| Authors. Published Year. Journal | Exclusion reason |
| --- | --- |
| Efficacy and Safety of Sodium-Glucose Cotransporter-2 Inhibitor in Diabetic Kidney Transplant Recipients: A Case-Control StudyIbrahim A.A.; Ali M.Y.; Mahgoub A.; Rahman R.M.A.; Abdelhalim M.; Hamdi A.F.; Ghonimi T.A.; Fouda T.A.; Hussain M.E.; Elshirbeny M.; Eltayeb F.B.; Elgaali M.; Arroub S.A.; Ibrahim R.A.; Abuhelaiqa E.; Hamad A.I.; Al-Malki H.A.; Alkadi M.M. 2023. Journal of the American Society of Nephrology | Abstract only |
| Comparative Safety Analysis of Empagliflozin in Type 2 Diabetes Mellitus Patients with Chronic Kidney Disease versus Normal Kidney Function: A Nationwide Cohort Study in KoreaJang H.Y.; Kim I.-W.; Oh J.M. 2023. Pharmaceutics | Wrong outcomes |
| Effect of dapagliflozin on COVID-19 infection and risk of hospitalizationSalgado-Barreira A.; Seijas-Amigo J.; Rodriguez-Manero M.; Pineiro-Lamas M.; Eiras S.; Cordero A.; Gonzalez-Juanatey J.R.; Figueiras A. 2023. Journal of Antimicrobial Chemotherapy | Wrong patient population |
| Efficacy and safety of empagliflozin-based quadruple therapy compared to insulin glargine-based therapy in poorly controlled type 2 diabetesKu E.; Jeon H.; Oh T. 2018Diabetologia | Abstract only |
| Effectiveness and safety of dapagliflozin in real-life patients: data from the DAPA-RWE Spanish multicentre studyMorales C.; Merino-Torres J.F.; Moreno-Moreno P.; Lainez M.; Tejado I.; Yoldi A.; Medina S.G.; Soto A.; Botana M.A.; Bellido V.; Caballero I. 2022Drugs in Context | No comparison groups |
| Does prescription of SGLT2i decrease the risk of COVID- 19 in adults with T2DM in the UK, compared to prescription of DPP4i: A CPRD population-based observational cohort studyTaylor O.A.; Mathur R. 2022. Pharmacoepidemiology and Drug Safety | Abstract only |
| Sodium glucose cotransporter 2 inhibitors and the risk of respiratory outcomes among patients with type 2 diabetes: A nationwide cohort studyJeong H.E.; Park S.; Noh Y.; Filion K.; Yu O.H.Y.; Jang S.H.; Shin J.-Y. 2022. Pharmacoepidemiology and Drug Safety | Abstract only |
| Assessment of empagliflozin add-on therapy to metformin and glimepiride in patients with inadequately controlled type-2 diabetes mellitusJawed B.; Qamar Abbas S.; Ahmed Z.; Andleeb S.; Ahmad S.A.; Asif M.; Akhter E.; Ahmed S.; Hussain M.W.; Iqbal A.; Ishaqui A.A. 2022. Pakistan Journal of Pharmaceutical Sciences | Wrong outcomes |
| Safety of Sodium-Glucose Cotransporter-2 Inhibitors in Patients with CKD and Type 2 Diabetes: Population-Based US Cohort StudyFu E.L.; D'andrea E.; Wexler D.J.; Patorno E.; Paik J.M. 2023. Clinical Journal of the American Society of Nephrology | Wrong outcomes |
| Dipeptidyl Peptidase-4 Inhibitors, Glucagon-like Peptide-1 Receptor Agonists, and Sodium-Glucose Cotransporter-2 Inhibitors and COVID-19 OutcomesForesta A.; Ojeda-Fernandez L.; Macaluso G.; Roncaglioni M.C.; Tettamanti M.; Fortino I.; Leoni O.; Genovese S.; Baviera M. 2023. Clinical Therapeutics | Wrong patient population |
| Investigating effectiveness and safety of sodium-glucose co-transporter 2 inhibitors in type 2 diabetes in Scotland: A real-world observational pharmacoepidemiology studyCaparrotta T.M.; Blackbourn L.A.K.; Colhoun H.M.; McKeigue P.M.; McGurnaghan S.J. 2023Diabetic Medicine | Abstract only |
| Empagliflozin reduces cardiorenal events, healthcare resource use and mortality in Sweden compared to dipeptidyl peptidase-4 inhibitors: Real world evidence from the Nordic EMPRISE studyNystrom T.; Toresson Grip E.; Gunnarsson J.; Casajust P.; Karlsdotter K.; Skogsberg J.; Ustyugova A. 2023Diabetes, Obesity and Metabolism | Wrong outcomes |
| Postcardiac Surgery Euglycemic Diabetic Ketoacidosis in Patients on Sodium-Glucose Cotransporter 2 InhibitorsAuerbach J.S.; Gershengorn H.B.; Aljure O.D.; Lamelas J.; Patel S.S.; Ferreira T.D.; Gonzalez L.A.; Cabrera J.L. 2023Journal of Cardiothoracic and Vascular Anesthesia | Wrong patient population. Not clear if T2D only |
| Comparison of the Efficacy and Safety of Various Oral Antidiabetic Drug Regimens Used for Type-2 Diabetes Mellitus-in a Tertiary Care Hospital in South DelhiMalik M.; Habib A.; Ahmad R.; Khanna S. 2023International Journal of Toxicological and Pharmacological Research | Wrong outcomes |
| Outpatient medications associated with protection from COVID-19 hospitalizationSandhu H.S.; Lambert J.; Steckler Z.; Park L.; Stromberg A.; Ramirez J.; Jeffrey Yang C.-F. 2023. PLoS ONE | Wrong patient population |
| Efficacy and Safety of Empagliflozin in Patients with Type 2 Diabetes Mellitus Fasting During Ramadan: A Real-World Study from BangladeshPathan M.D.F.; Akter N.; Selim S.; Saifuddin M.; Qureshi N.K.; Kamrul-Hasan A.B.M.; Hannan M.A.; Ahmed M.A.U.; Mustari M.; Chakraborty A.K. 2022. Diabetes, Metabolic Syndrome and Obesity | Wrong outcomes |
| Empagliflozin cardiovascular and renal effectiveness and safety compared to dipeptidyl peptidase-4 inhibitors across 11 countries in Europe and Asia: Results from the EMPagliflozin compaRative effectIveness and SafEty (EMPRISE) studyKarasik A.; Lanzinger S.; Chia-Hui Tan E.; Yabe D.; Kim D.J.; Sheu W.H.-H.; Melzer-Cohen C.; Holl R.W.; Ha K.H.; Khunti K.; Zaccardi F.; Subramanian A.; Nirantharakumar K.; Nystrom T.; Niskanen L.; Linnemann Jensen M.; Hoti F.; Klement R.; Deruaz-Luyet A.; Kyaw M.H.; Koeneman L.; Vistisen D.; Carstensen B.; Halvorsen S.; Langslet G.; Fazeli Farsani S.; Patorno E.; Nunez J. 2023. Diabetes and Metabolism | Wrong outcomes |
| Efficacy And Safety Of Sodium Glucose Co- Transporter 2 Inhibitors In Type 2 Diabetes Mellitus PatientsMani P.; Vohra A.; Jain S.; Chandrabhan; Mathur R. 2022. European Journal of Molecular and Clinical Medicine | Abstract only |
| Association of Sodium-Glucose Cotransporter 2 Inhibitor vs Dipeptidyl Peptidase-4 Inhibitor Use With Risk of Incident Obstructive Airway Disease and Exacerbation Events Among Patients With Type 2 Diabetes in Hong KongAu P.C.M.; Tan K.C.B.; Lam D.C.L.; Cheung B.M.Y.; Wong I.C.K.; Kwok W.C.; Sing C.-W.; Cheung C.-L. 2023. JAMA network open | Wrong outcomes |
| The Efficacy and Safety of SGLT2 Inhibitor in Diabetic Kidney Transplant RecipientsLim J.-H.; Kwon S.; Jeon Y.; Kim Y.H.; Kwon H.; Kim Y.S.; Lee H.; Kim Y.-L.; Kim C.-D.; Park S.-H.; Lee J.S.; Yoo K.D.; Son H.E.; Jeong J.C.; Lee J.; Lee J.P.; Cho J.-H. 2022. Transplantation | Wrong outcomes |
| Risk of infections in patients with NAFLD and Type 2 Diabetes under treatment with SGLT2 inhibitors and relationship with liver outcomes: A retrospective case-control studyBanares J.; Manzano-Nunez R.; Prio A.; Rivera-Esteban J.; Camps-Relats L.; Villarejo A.; Ruiz-Ortega L.; Pons M.; Ciudin A.; Salcedo M.T.; Vargas V.; Genesca J.; Pericas J.M. 202. 2Frontiers in Endocrinology | Wrong outcomes |
| Sodium-Glucose Cotransporter 2 Inhibitors, Glucagon-Like Peptide-1 Receptor Agonists, and Dipeptidyl Peptidase-4 Inhibitors, and Risk of HospitalizationLyu B.; Grams M.E.; Chang A.; Inker L.A.; Coresh J.; Shin J.-I. 2022American Journal of Cardiology | Wrong outcomes |
| Clinical benefits of empagliflozin in very old patients with type 2 diabetes hospitalized for acute heart failurePerez-Belmonte L.M.; Sanz-Canovas J.; Millan-Gomez M.; Osuna-Sanchez J.; Lopez-Sampalo A.; Ricci M.; Jimenez-Navarro M.; Lopez-Carmona M.D.; Bernal-Lopez M.R.; Barbancho M.A.; Lara J.P.; Gomez-Huelgas R. 2022Journal of the American Geriatrics Society | Abstract only |
| Sodium-Glucose Cotransporter-2 Inhibitors Are Associated With Lower Risks of Hospitalization for Heart Failure and All-Cause Mortality in Patients With Diabetes Mellitus and CancerChiang C.H.; Ma K.; Peng C.-Y.; Yuanping H.; Horng C.-S.; Chen C.-Y.; Chang Y.-C.; See X.Y.; Chen Y.-J.; Wang S.-S.; Suero Abreu G.A.; Peterson L.R.; Thavendiranathan P.; Armand P.; Peng C.-M.; Shiah H.-S.; Neilan T.G. 2022. Circulation | Abstract only |
| Real-World Safety of SGLT2 Inhibitors, DPP-4 Inhibitors, and GLP-1 Agonists in US Veterans With and Without Chronic Kidney DiseaseNarasaki Y.; Kovesdy C.; You A.; Potukuchi P.; Dashputre A.; Sumida K.; Thomas F.; Streja E.; Kalantar-Zadeh K.; Rhee C. 2022. American Journal of Kidney Diseases | Abstract only |
| Association of antidiabetic therapies with lower extremity amputation, mortality and healthcare cost from a nationwide retrospective cohort study in TaiwanChang H.-Y.; Chou Y.-Y.; Tang W.; Chang G.-M.; Hsieh C.-F.; Singh S.; Tung Y.-C. 2021. Scientific reports | Wrong patient population. Not clear if T2D only |
| Real-world evidence for long-term safety and effectiveness of ipragliflozin in treatment-naive versus non-naive Japanese patients with type 2 diabetes mellitus: subgroup analysis of a 3-year post-marketing surveillance study (STELLA-LONG TERM)Maegawa H.; Tobe K.; Nakamura I.; Uno S. 2021. Diabetology International | No comparison groups |
| Association between glucagonlike peptide 1 receptor agonist and sodium-glucose cotransporter 2 inhibitor use and covid-19 outcomesKahkoska A.R.; Abrahamsen T.J.; Alexander G.C.; Bennett T.D.; Chute C.G.; Haendel M.A.; Klein K.R.; Mehta H.; Miller J.D.; Moffitt R.A.; Stuurmer T.; Kvist K.; Buse J.B. 2021. Diabetes Care | Wrong patient population |
| Sodium-glucose co-transporter 2 inhibitors reduce hepatic events in diabetic patients with chronic hepatitis BLiang L.Y.; Wong V.W.-S.; Hui V.W.-K.; Yip T.C.-F.; Tse Y.-K.; Lui G.C.-Y.; Chan H.L.-Y.; Wong G.L.-H. 2021. GastroHep | Wrong patient population. Not clear if T2D only |
| Comparable COVID-19 outcomes with current use of GLP-1 receptor agonists, DPP-4 inhibitors or SGLT-2 inhibitors among patients with diabetes who tested positive for SARS-CoV-2Israelsen S.B.; Pottegard A.; Sandholdt H.; Madsbad S.; Thomsen R.W.; Benfield T. 2021Pharmacoepidemiology and Drug Safety | Abstract only |
| Use of oral anti-diabetic drugs and risk of hospital and intensive care unit admission for infectionsRim J.G.; Gallini J.; Jasien C.; Cui X.; Phillips L.; Trammell A.; Sadikot R. 2021Journal of Investigative Medicine | Wrong outcomes |
| Sodium-glucose cotransporter-2 inhibitors at discharge from cardiology hospitalization department: Decoding a new clinical scenarioRozado J.; Iglesias D.G.; Soroa M.; Junco-Vicente A.; Barja N.; Adeba A.; Vigil-Escalera M.; Alvarez R.; Saura F.T.; Capin E.; Garcia L.; Rodriguez M.L.; Calvo D.; Moris C.; Delgado E.; de la Hera J.M. 2020Journal of Clinical Medicine | Wrong outcomes |
| Real-world evidence on sodium-glucose cotransporter-2 inhibitor use and risk of Fournier's gangreneYang J.Y.; Wang T.; Pate V.; Buse J.B.; Sturmer T. 2020BMJ Open Diabetes Research and Care | Wrong patient population. Not clear if T2D only |
| A tree-based statistic data mining approach to assess the post-marketing safety of new glucose-lowering medications in routine careFralick M.; Kulldorff M.; Redelmeier D.; Wang S.V.; Vine S.M.; Schneeweiss S.; Patorno E. 2020Pharmacoepidemiology and Drug Safety | Abstract only |
| A novel data mining approach to detect adverse events of new diabetes drugs in routine careFralick M.; Kulldorff M.; Wang S.; Schneeweiss S.; Redelmeier D.; Patorno E. 2020Diabetes | Abstract only |
| USE OF ORAL ANTI-DIABETIC DRUGS AND RISK OF HOSPITAL AND ICU ADMISSIONS FOR INFECTIONSRim J.; Gallini J.; Jasien C.; Cui X.; Phillips L.; Sadikot R.; Trammell A. 2020Chest | Abstract only |
| Increased risk of mycotic infections associated with sodium-glucose co-transporter 2 inhibitors: a prescription sequence symmetry analysisAdimadhyam S.; Schumock G.T.; Calip G.S.; Smith Marsh D.E.; Layden B.T.; Lee T.A. 2019British Journal of Clinical Pharmacology | Wrong outcomes |
| Assessing the risk of gout with sodium glucose co-transporter-2 inhibitors: A population-based cohort studyFralick M.; Chen S.; Patorno E.; Kim S.C. 2019Arthritis and Rheumatology | Abstract only |
| SGLT-2 inhibitors and the risk of community-acquired pneumonia in patients with type-2 diabetesBrunetti V.; Reynier P.; Azoulay L.; Yu O.H.Y.; Ernst P.; Platt R.W.; Filion K.B. 2019Pharmacoepidemiology and Drug Safety | Abstract only |
| Short-term outcomes of patients with type 2 diabetes mellitus treated with canagliflozin compared with sitagliptin in a real-world settingShao Y.L.; Yee K.H.; Koh S.K.; Wong Y.F.; Yeoh L.Y.; Low S.; Sum C.F. 2018Singapore Medical Journal | Wrong outcomes |
| Association of GLP-1 Receptor Agonists with Chronic Obstructive Pulmonary Disease Exacerbations among Patients with Type 2 Diabetes.Foer, Dinah; Strasser, Zachary H; Cui, Jing; Cahill, Katherine N; Boyce, Joshua A; Murphy, Shawn N; Karlson, Elizabeth W2023. American journal of respiratory and critical care medicine | Wrong outcomes |
| Comparative effectiveness of sodium-glucose co-transporter-2 inhibitors and dipeptidyl peptidase-4 inhibitors on liver function in patients with type 2 diabetes in Japan: A real-world data analysis.Takahashi, Hirokazu; Asakawa, Keiko; Kosakai, Yoshinori; Lee, Takumi; Rokuda, Mitsuhiro2024. Diabetes, obesity & metabolism | Wrong outcomes |
| Abstract #1603090: Real World Use, Effectiveness, and Safety of SGLT2 inhibitors in Patients With Type 2 Diabetes: Experience in A Tertiary Care Hospital in QatarElgamal M.; Moursi M.; Ibrahim A.; Elzouki A.-N. 2023. Endocrine Practice | Abstract only |
| IMPACT OF USE OF SODIUM-GLUCOSE COTRANSPORTER 2 INHIBITORS ON DEVELOPMENT OF LIVER-RELATED COMPLICATIONS IN PATIENTS WITH TYPE 2 DIABETES: A TERRITORYWIDE COHORT STUDYLi G.; Leung Y.; Yam T.-F.; Wong G.L.-H.C.; Wong V.W.-S.; Yip T.C.-F. 2023. Hepatology | Abstract only |
| Comparing the effectiveness of glucose-lowering agents: real-world data to emulate a four-arm target trialVural Keskinler M.; Telci Caklili O.; Oguz A. 2023The Lancet Diabetes and Endocrinology | Letter only |
| Comparing the effectiveness of glucose-lowering agents: real-world data to emulate a four-arm target trialAhmed A.; Anker S.D.; Butler J.; Bakris G.L.; Bhatt D.L.; Fonarow G.C.; Packer M. 2023. The Lancet Diabetes and Endocrinology | Letter only |
| Elibol A, Eren D, Erdoğan MD, Elmaağaç M, Dizdar OS, Çelik İ, Günal Aİ. Factors influencing on development of COVID-19 pneumonia and association with oral anti-diabetic drugs in hospitalized patients with diabetes mellitus. Prim Care Diabetes. 2021 Oct;15(5):806-812. doi: 10.1016/j.pcd.2021.08.001. Epub 2021 Aug 4. PMID: 34376379; PMCID: PMC8332925. | Wrong study design (cross-sectional study) |

# Supplementary 5 – Additional details for each individual study included

| Outcome | Firth author, year Country | Mean age, years (SD) | Female n, % | Glycated haemoglobin, % | Body mass index, kg/m2 | Diabetes duration, years | Infection-outcome as primary analysis Y/N |
| --- | --- | --- | --- | --- | --- | --- | --- |
| Confirmed or suspected COVID-19,  Confirmed COVID-19 | Sainsbury, 2021  United Kingdom | 60.6 (10.8) | 2864, 37.3% | NR | 33 | 10.30 | Y |
|  |  | 62.6 (10.4) | 2899, 37.8% | NR | 32.20 | 10.60 |  |
| COVID-19 Hospitalisation | Piarulli, 2023 Italy | NR | NR | NR | NR | NR | Y |
|  |  | NR | NR | NR | NR | NR |  |
| Incident severe COVID-19 | Ferrannini, 2023 Sweden | 66 | 12711, 32.4% | NR | NR | NR | Y |
|  |  | 72 | 133239, 43.7% | NR | NR | NR |  |
| COVID-19 case fatality rate | Shestakova, 2022 Russia | NR | NR | NR | NR | NR | Y |
|  |  | NR | NR | NR | NR | NR |  |
| COVID-19 Mortality | Araldi, 2023 United Kingdom (preprint) | NR | NR | NR | NR | NR | Y |
|  |  | NR | NR | NR | NR | NR |  |
| COVID-19 All-cause mortality, COVID-19 Pneumonia, COVID-19 Sepsis, | Lim, 2024 South Korea | NR | 5515, 47.9% | NR | NR | NR | N |
|  |  | NR | 5388, 46.8% | NR | NR | NR |  |
| COVID-19 Mortality | Kunthi, 2022 United Kingdom | 69 (13) | 63, 27% | NA | 30.4 | NR | N |
|  |  | 73 (14) | 1,094, 39% | NA | 29.3 | NR |  |
| COVID-19 In-hospital death, Hospitalisation | Min, 2022 United States | 62 (11) | 0.45 | 7.9 | 31.3 | NR | Y |
|  |  | 62 (11) | 0.45 | 7.9 | 31.3 | NR |  |
| COVID-19 ICU, intubation, death, Hospitalisation | Hsin-Chie, 2022 United States of America | NR | NR | NR | NR | NR | Y |
|  |  | NR | NR | NR | NR | NR |  |
| COVID-19 In-hospital death | Pérez-Belmonte, 2020 Spain | 69.8 (4.2) | 12, 35.3% | NR | NR | NR | Y |
|  |  | 73.8 (6.0) | 13, 38.2% | NR | NR | NR |  |
| COVID-19 In-hospital death | Ramos-Rincón, 2021 Spain | 86 (6.2) | 166, 43.2% | NR | NR | NR | Y |
|  |  | 85.8 (6.2) | 206, 51.0% | NR | NR | NR |  |
| COVID-19 In-hospital death | Monda, 2023 Italy | 61 (8) | 154, 47% | 7.70 | 29.80 | NR | Y |
|  |  | 61 (12) |  | 7.8 | 30.1 | NR |  |
| COVID-19 Mortality | Khunti, 2021 United Kingdom | NR | 104435, 39·2% | NR | NR | NR | Y |
|  |  | NR | 104435, 39·2% | NR | NR | NR |  |
| COVID-19 Mortality, Severe COVID-19 outcomes | Ouchi, 2022 Spain | NR | NR | NR | NR | NR | Y |
|  |  | 71.07 (14.44) | 2979, 48.3% | NR | NR | 6 |  |
| COVID-19 Mortality, Hospitalisation | Mannucci, 2022 Italy | 64.2 | 71, 30.9% | NR | NR | 11 | Y |
|  |  | NR | NR | NR | NR | NR |  |
| Hospitalisation for HCAP | Brunetti, 2021 United Kingdom | 58.1 (9.5) | 641, 63.4% | 9.4 | NR | 8 | Y |
|  |  | 63.0 (11.9) | 3463, 62.4% | 8.9 | NR | 6.5 |  |
| Pneumonia, Pneumonia-mortality | Au, 2022 Hong Kong | 61.41 (9.83) | 2913, 43.7% | 8.59 | NR | 9 | Y |
|  |  | 61.52 (10.05) | 11824, 44.4% | 8.53 | NR | 9.1 |  |
| Pneumonia | Jeong, 2023 South Korea | 53.9 (13.0) | 83719, 40.7% | NR | NR | NR | Y |
|  |  | 53.7 (13.4) | 82980, 40.4% | NR | NR | NR |  |
| Pneumonia, Pneumonia-mortality, Sepsis, Sepsis-death | Wu, 2022 Hong Kong | 59 (11) | 4058, 37.90% | 8.3 | NR | NR | Y |
|  |  | 60 (11) | 7207, 39.42% | 8.2 | NR | NR |  |
| Influenza or pneumonia, Respiratory infection  Acute upper respiratory infection | Park, 2023 South Korea | NR | 145, 48.3% | NR | NR | NR | Y |
|  |  | NR | 242, 44.1% | NR | NR | NR |  |
| Pneumonia Sepsis COPD | Tan, 2024 Australia | 62.5 | 59165, 59.4% | NR | NR | 9.3 | N |
|  |  | 65.2 | 107272, 57.6% | NR | NR | 7.7 |  |
| ICU admissions for sepsis, Mortality due to Infections | Ng, 2023 Hong Kong | 58.9 (10.8) | 3726, 36.1% | 8.6 | NR | 7.4 | Y |
|  |  | 59.8 (11.2) | 6830, 38.7% | 8.6 | NR | 7.5 |  |
| Sepsis/septic shock | Hu, 2024 Taiwan | 59.46 (12.29) | 131323, 43.02% | NR | NR | NR | Y |
|  |  | 59.62 (12.86) | 130442, 42.73% | NR | NR | NR |  |
| Fournier’s gangrene or necrotizing fasciitis | Petruski-Ivleva, 2020 United States of America | 54.3 (9.1) | 1366, 39.1% | NR | NR | NR | Y |
|  |  | 54.3 (9.9) | 1355, 38.8% | NR | NR | NR |  |
|  |  | 53.5 (9.5) | 1064, 48.1% | NR | NR | NR |  |
|  |  | 53.5 (10) | 1064, 48.1% | NR | NR | NR |  |
|  |  | 53.9 (9.3) | 1101, 40.3% | NR | NR | NR |  |
|  |  | 53.7 (9.8) | 1091, 39.9% | NR | NR | NR |  |
| Hospitalisation for Fournier’s gangrene | Wang, 2020 United States of America | 56.2 (10.9) | 15, 6.9% | NR | NR | NR | Y |
|  |  | 56.4 (10.5) | 90, 6.9% | NR | NR | NR |  |
| Time to first COPD exacerbation: Moderate  Number of COPD exacerbations: Moderate | Pradhan, 2022 United Kingdom | 62.9 (9.0) | 1227, 41.5% | NR | NR | 9.1 | Y |
|  |  | 62.7 (9.1) | 4551, 42% | NR | NR | 8.5 |  |
| New-onset infective  endocarditis | Chou, 2023 (Preprint) Hong-Kong | 57.9 (11.3) | 11638, 40.44% | 8.3 | NR | 6.5 | Y |
|  |  | 59.2 (11.1) | 12034, 41.82% | 8.2 | NR | 6.4 |  |
| Infectious keratitis | Tsai, 2024 Taiwan | NR | 14419, 34.56% | NR | NR | NR | Y |
|  |  | NR | 14330, 34.34% | NR | NR | NR |  |
| Abbreviations: COVID-19: Coronavirus Disease 2019; COPD: Chronic Obstructive Pulmonary Disease; NR: Not Reported; SD: Standard Deviation; Y/N: Yes/No | | | | | | | |

# Supplementary 6 – Risk of bias assessments

| Study ID | Outcome | Bias due to confounding (baseline confounding, time-varying confounding) | Bias in selection of participants into the study | Bias in classification of interventions | Bias due to deviations from the intended intervention | Bias due to missing data | Bias in measurement of outcomes | Bias in selection of the reported result | Overall risk of bias |
| --- | --- | --- | --- | --- | --- | --- | --- | --- | --- |
| Monda 2023 | COVID-19 mortality | Low risk; PS matched based on baseline covariates such as demographics, comorbidities, glycaemic control, previous hospitalisation; no need to assess risk of time-varying confounding (ITT analyses) | Moderate risk; prevalent user design included potential new users and prevalent users (treatment decision design implemented); all included subjects have also signed an informed consent form for being included in the study (may be non-differential) | Low risk; both intervention and active comparator arm requires at least 2 dispensations during defined exposure ascertainment period before index date | Low risk; ITT analysis conducted | No information; authors did not report data completeness | Low risk; in-hospital death was recorded during the relatively short follow-up | Low risk; there was an a priori study protocol; there was no indication of selective reporting based on intervention, outcome, multiple analyses, or different subgroups | Moderate risk of bias |
| Sainsbury 2021 | Confirmed or suspected COVID-19 | Low risk; PS matched based on baseline covariates such as sociodemographic, lifestyle and metabolic profile, comorbidities, diabetes severity and duration, history of prescription drugs; negative control outcome used to assess confounding bias; no need to assess risk of time-varying confounding (ITT analyses) | Moderate risk; prevalent user design included potential new users and prevalent users (treatment decision design implemented) | Low risk; both intervention and active comparator arm requires a record of prescription that covers beyond the index date | Low risk; ITT analysis conducted | Low risk; low prevalence of missing covariate data; unclear how missing data were handled | Low risk; individuals were censored upon leaving GP practice, or when ceased contributing to the database; negative control outcome used to assess surveillance bias | Moderate risk of bias; no a priori protocol, but there was no indication of selective reporting based on intervention, outcome, multiple analyses, or different subgroups | Moderate risk of bias |
| Khunti 2021 | COVID-19 mortality | Low risk; PS matched based on an extensive list of baseline covariates such as sociodemographic, diabetes severity and duration, smoking status, BMI, comorbidities, history of prescription drugs; no need to assess risk of time-varying confounding (ITT analyses) | Serious risk; users are compared to non-users of the exposure drug, leading to risk of healthy user bias | Low risk; both intervention and active comparator arm requires at least 1 dispensation during defined exposure ascertainment period before index date | Low risk; ITT analysis conducted | Moderate risk; only individuals with missing information on sex were excluded; used the missing category approach; prevalence of missing data is generally low (except ethnicity); | Low risk; COVID-related mortality was recorded during short follow-up time (~0.5 year); however, censoring requirements were unclear | Moderate risk of bias; no a priori protocol, but there was no indication of selective reporting based on intervention, outcome, multiple analyses, or different subgroups | Serious risk of bias |
| Piarulli 2023 | COVID-19 hospitalisation | Low risk; logistic regression model controlled for baseline covariates such as demographics, diabetes duration, HbA1c, BMI, comorbidities, antidiabetic therapy and other comedications; no need to assess risk of time-varying confounding | Serious risk; cohort study design with unclear temporal relationship between exposure and outcome | Serious risk; intervention not clearly defined; unclear exposure ascertainment period | Low risk; not relevant for study design | No information; authors did not report data completeness | Low risk; COVID-related hospitalisations were recorded in the database | Moderate risk of bias; no a priori protocol, but there was no indication of selective reporting based on intervention, outcome, multiple analyses, or different subgroups | Serious risk of bias |
| Ferrannini 2023 | Incident severe COVID-19 | Low risk; PS matched based on baseline covariates such as demographics, comorbidities, glycaemic control, previous hospitalisation; no need to assess risk of time-varying confounding (ITT analyses) | Serious risk; users are compared to non-users of the exposure drug, leading to risk of healthy user bias | Serious risk; immortal time bias as exposure ascertainment was conducted after the outcome; prevalent user design | Low risk; ITT analysis conducted | Low risk; low prevalence of missing covariate data; individuals with missing data were excluded (complete case approach) | Low risk; COVID-related hospitalisations were recorded in the national databases | Moderate risk; no a priori protocol, but there was no indication of selective reporting based on intervention, outcome, multiple analyses, or different subgroups | Serious risk of bias |
| Mannucci 2022 | COVID-19 mortality | Moderate risk; logistic regression model controlled for baseline covariates such as demographics, diabetes duration, comedications, comorbidities; no adjustment for laboratory test and BMI; no need to assess risk of time-varying confounding | Serious risk; users are compared to non-users of the exposure drug, leading to risk of healthy user bias | Moderate risk; individuals with interventions were compared to those without, however, the presence of intervention was determined using a varying window from a set calendar date till the outcome | Low risk; not relevant as study has no follow-up period | No information; authors did not report data completeness | Low risk; COVID-related hospitalisations were recorded in the regional databases | Moderate risk; no a priori protocol, but there was no indication of selective reporting based on intervention, outcome, multiple analyses, or different subgroups | Serious risk of bias |
| Ouchi 2022 | COVID-19 mortality and severe outcomes | Low risk; logistic regression model controlled for baseline covariates such as demographics, obesity and smoking status, HbA1c, comorbidities including diabetes, comedications; no need to assess risk of time-varying confounding (ITT analyses) | Moderate risk; prevalent user design included potential new users and prevalent users (treatment decision design implemented) | Low risk; both intervention and active comparator arm requires at least 1 dispensation during defined exposure ascertainment period before index date | Low risk; ITT analysis conducted | Moderate risk; large proportion of missing values for HbA1c (46.5%) | Low risk; COVID-related outcomes were recorded in the regional databases | Moderate risk; no a priori protocol, but there was no indication of selective reporting based on intervention, outcome, multiple analyses, or different subgroups | Moderate risk of bias |
| Shestakova 2022 | COVID-19 Case fatality rate | Low risk; logistic regression model controlled for demographics, diabetes duration, BMI, HbA1c, renal function, other laboratory tests, comorbidities, COVID-19 vaccinations, COVID-19 antivirals; no need to assess risk of time-varying confounding | Serious risk; users are compared to non-users of the exposure drug, leading to risk of healthy user bias | Moderate risk; unclear exposure ascertainment period (only defined as pre index date) | Low risk; ITT analysis conducted | No information; authors did not report data completeness | Low risk; relatively short follow-up period and COVID-related mortality reported in national database | Moderate risk; no a priori protocol, but there was no indication of selective reporting based on intervention, outcome, multiple analyses, or different subgroups | Serious risk of bias |
| Wang 2020 | Hospitalisation for Fournier’s gangrene | Low risk; logistic regression model controlled for baseline covariates such as demographics, comorbidities, diabetes complications, insulin use, previous hospitalisations and comedications; no adjustment laboratory test and BMI; no need to assess risk of time-varying confounding | Moderate risk; prevalent user design included potential new users and prevalent users | Low risk; both intervention and active comparator groups require supply of oral AHA that included the index date | Low risk; not relevant for case-control study design | No information; authors did not report data completeness | Low risk; used outcome definition as a previous study; nested case-control study with follow-up from cohort entry until event, death, disenrollment from health plan or end of data period, whichever occurs first | Moderate risk; no a priori protocol, but there was no indication of selective reporting based on intervention, outcome, multiple analyses, or different subgroups | Low risk of bias |
| Petruski-Ivleva 2020 | Fournier’s gangrene or necrotizing fasciitis | Moderate risk; PS matching with model considering demographics, comorbidities, previous UTI and diseases of the GU system, hospitalisations for bacterial infections, obesity status, comedications; no adjustment for diabetes severity/duration and laboratory tests inc. HbA1c; only baseline confounders covariates considered, hence low risk of time-varying confounding | Low risk; active comparator new user design | Low risk; all cohorts were defined as new users using a similar exposure washout period; however, unclear exposure definition (1 v. multiple dispensations) | Moderate risk; individuals were censored upon drug discontinuation or initiation of counterpart drug; unknown risk of differential drug discontinuation | No information; authors did not report data completeness | Low risk; multiple outcome definitions were used as sensitivity analyses; people were censored on discontinued of drug, switch in therapy, end of enrolment in health plan or end of data period | Moderate risk; no a priori protocol, but there was no indication of selective reporting based on intervention, outcome, multiple analyses, or different subgroups | Moderate risk |
| Wu 2022 | Pneumonia, pneumonia-mortality, sepsis, sepsis-mortality | Low risk; PS matching with model considering demographics, calendar year, diabetes duration, diabetes complications, risk factors for outcomes, comorbidities, lifestyle factors, antidiabetic medications and other comedications, HbA1c and renal function at baseline; negative control outcome used to assess unmeasured confounding; no need to assess risk of time-varying confounding | Low risk; active comparator new user design | Low risk; both cohorts were defined as new users using a similar exposure washout period | Low risk; ITT analysis conducted | No information; authors did not report data completeness | Low risk; individuals were censored upon outcome, death or last date of data collection, whichever came first | Moderate risk; no a priori protocol, but there was no indication of selective reporting based on intervention, outcome, multiple analyses, or different subgroups | Low risk of bias |
| Brunetti 2021 | Pneumonia | Low risk; Cox regression model adjusted for demographics, calendar year, comorbidities, previous Dx of pneumonia, BP, HbA1c, lifestyle factors, diabetes severity/duration and complications, and healthcare utilisation; no need to assess time-varying confounding | Moderate risk; prevalent user design included potential new users and prevalent users (treatment decision design implemented) | Low risk; both cohorts were defined as current users using prescription supplies that overlapped with index date | Low risk; ITT analysis conducted | Low risk; multiple imputation conducted for missing covariate information; prevalence of missing data was low | Low risk; individuals were censored upon outcome, competing outcome (hospital-acquired pneumonia), end of registration with primary health care record system, or end of data period, whichever came first | Low risk; there was an a priori study protocol; there was no indication of selective reporting based on intervention, outcome, multiple analyses, or different subgroups | Moderate risk of bias |
| Au 2021 | Pneumonia | Low risk; PS matching with model considering demographics, comedications, use of glucose-lowering agents, vaccination history, medical history, diabetes complications, healthcare utilisation, HbA1c, renal function; no need to assess risk of time-varying confounding | Low risk; prevalent new user design compared users of the intervention to users of active comparator based on the length of previous exposure to the comparator | Low risk; both cohorts were defined by using clear definition | Low risk; ITT analysis conducted (PP analysis conducted as a sensitivity analysis) | No information; authors did not report data completeness | Low risk; individuals were censored upon outcome, death, or end of data period, whichever came first | Moderate risk; no a priori protocol, but there was no indication of selective reporting based on intervention, outcome, multiple analyses, or different subgroups | Low risk of bias |
| Jeong 2023 | Pneumonia | Moderate risk; Cox regression model adjusted for demographics, comorbidities, comedications, level of antidiabetic treatment, and baseline healthcare utilisation; no adjustment based on HbA1c and laboratory tests; only baseline confounders covariates considered, hence low risk of time-varying confounding | Low risk; active comparator new user design | Low risk; both cohorts were defined by using clear definition | Moderate risk; individuals were censored upon drug discontinuation or initiation of counterpart drug; unknown risk of differential drug discontinuation | No information; authors did not report data completeness | Low risk; individuals were censored at the earliest occurrence of drug discontinuation, switching to comparator drug, in-hospital death or end of study. | Moderate risk; no a priori protocol, but there was no indication of selective reporting based on intervention, outcome, multiple analyses, or different subgroups | Moderate risk of bias |
| Park 2023 | Pneumonia | Moderate risk; PS matching with model considering demographics, comedications, comorbidities; no adjustment based on HbA1c, diabetes duration/severity, and laboratory tests; only baseline confounders covariates considered, hence low risk of time-varying confounding | Moderate risk; prevalent user design included potential new users and prevalent users | Low risk; both cohorts were defined using concomitant use of intervention or comparator drug (at least 30-day supply) with insulin | Moderate risk; individuals were censored upon drug discontinuation or initiation of counterpart drug; unknown risk of differential drug discontinuation | No information; authors did not report data completeness | Low risk; individuals were censored at the earliest occurrence of outcome event, drug discontinuation, death or end of study period | Moderate risk; no a priori protocol, but there was no indication of selective reporting based on intervention, outcome, multiple analyses, or different subgroups | Moderate risk of bias |
| Ng 2023 | Sepsis | Low risk; PS matching with model considering demographics, comedications, comorbidities, duration of diabetes, previous ICU admission, HbA1c and baseline renal function; no need to assess time-varying confounding | Low risk; active comparator new user design | Low risk; both cohorts were defined by using clear definition | Low risk; ITT analysis conducted | Low risk; complete case approach and multiple imputation method were used to assess the robustness of results; only HbA1ca and eGFR (renal function) had missing data | Low risk; individuals were censored upon outcome, death, or end of data period, whichever came first | Moderate risk; no a priori protocol, but there was no indication of selective reporting based on intervention, outcome, multiple analyses, or different subgroups | Low risk of bias |
| Pradhan 2022 | infectious COPD exacerbation | Low risk; PS stratification conducted with model controlling for demographics, BMI, smoking status, diabetes severity/duration, comedications, diabetes complications, HbA1c, previous respiratory events, healthcare utilisation; only baseline confounders covariates considered, hence low risk of time-varying confounding | Low risk; active comparator new user design | Low risk; both cohorts were defined by using clear definition | Low risk; individuals were censored upon drug discontinuation or initiation of counterpart drug; unknown risk of differential drug discontinuation; used different outcome definitions as sensitivity analyses | Low risk; variables with missing values (BMI, smoking status, HbA1c, and other laboratory tests) were modelled with an unknown category; low prevalence of missing data | Low risk; individuals were censored at the earliest occurence of outcome event, drug discontinuation, switching therapy, death, end of registration with database, or end of study period | Moderate risk; no a priori protocol, but there was no indication of selective reporting based on intervention, outcome, multiple analyses, or different subgroups | Low risk of bias |
| Chou 2023 | Infectious endocarditis | Low risk; PS matching with model considering demographics, HbA1c, cholesterol levels, diabetes duration, comorbidities, comedications, use of financial aids; only baseline confounders covariates considered, hence low risk of time-varying confounding | Moderate risk; active comparator design; however, unclear whether new user design was implemented | Serious risk; intervention not clearly defined; unclear exposure ascertainment period | Moderate risk; individuals were censored upon drug discontinuation or initiation of counterpart drug; unknown risk of differential drug discontinuation | Low risk; multiple imputation conducted for missing covariate information | Low risk; individuals were censored at the earliest occurence of outcome event, drug discontinuation, switching therapy, death, or end of study period | Moderate risk; no a priori protocol, but there was no indication of selective reporting based on intervention, outcome, multiple analyses, or different subgroups | Serious risk of bias |
| Araldi 2023 | COVID-19 related mortality (and many others) | Moderate risk; nearest neighbour covariate matching using covariates such as ??? ; no need to assess time-varying confounding | Serious risk; users of intervention were compared to non-users | Low risk; both cohorts were defined by using clear definition | Low risk; ITT analysis conducted | Low risk; only individuals with clinical data and prescription data were included in the study; complete case approach | Low risk; individuals were followed up until censoring for death from any cause (data from national death registry) | Moderate risk; no a priori protocol, but there was no indication of selective reporting based on intervention, outcome, multiple analyses, or different subgroups | Serious risk of bias |
| Lim, 2024 | COVID-19 related mortality  pneumonia  Sepsis | Moderate risk; PS matched based on baseline covariates such as age; female sex; and diagnosis of infectious gastrointestinal, respiratory, endocrinal, cardiac or malignant disease. No adjustment for: diabetes duration, HbA1c, BMI. | Serious risk: prevalent user design, users of intervention were compared to non-user | Low risk; both cohorts were defined by using clear definition: intervention cohort needed a prescription claim 6 months before their COVID-19 diagnosis | Low risk; ITT analysis conducted | No information; authors did not report data completeness | Low risk: patients were follow-up within 90 days for several clinical events defined. The time was defined based on a systematic review | Moderate risk; no a priori protocol, but there was no indication of selective reporting based on intervention, outcome, multiple analyses, or different subgroups | Serious risk of bias |
| Khunti, 2022 | COVID-19 related mortality | Low risk; logistic regression model controlled for baseline covariates such as age, sex, ethnicity, admission blood glucose level, insulin administration, micro- and macrovascular disease | Serious risk: prevalent user design, users of intervention were compared to non-user | Low risk; both cohorts were defined by using clear definition: defined as record or absence of record of SGLT2i use prior to hospital admission. | Low risk; ITT analysis conducted | Moderate risk: authors reported having a number of patients  with missing data for some variables. The group these patients belonged to was not reported | Low risk: An audit data collection form was provided to each participating centre, where pseudonymized  data were held on secure servers | Moderate risk; no a priori protocol, but there was no indication of selective reporting based on intervention, outcome, multiple analyses, or different subgroups | Serious risk of bias |
| Min, 2022 | COVID-19 related mortality  and hospitalisation | Low risk; PS weighting with matching weights and additional adjustment for all covariates age, sex, race, Hispanic ethnicity, SDI quintile, body mass index, systolic blood pressure, diastolic blood pressure, creatinine, baseline Exhauster comorbidities, baseline medications, baseline inpatient encounters, baseline outpatient encounters, baseline ED encounters, and baseline outpatient medications | Serious risk: prevalent user design, users of intervention were compared to non-user | Low risk; both cohorts were defined by using clear definition: required to have at least one prescription for metformin and at least one prescription for a non-insulin antidiabetic drug in the baseline (pre index) year. Exposure to four classes of antidiabetic drugs, were defined based on having at least one prescription in the baseline year | Low risk; ITT analysis conducted | No information; authors did not report data completeness | Low risk; individuals were followed up until outcome or end of follow-up date. COVID-19 hospitalisation was defined by a discharge diagnosis code for COVID-19 and/or a positive COVID-19 test result either during or 2 weeks prior to the hospitalisation. In-hospital death during a COVID-19 hospitalisation was defined as a secondary outcome. | Moderate risk; no a priori protocol, but there was no indication of selective reporting based on intervention, outcome, multiple analyses, or different subgroups | Serious risk of bias |
| Hsin-Chieh, 2022 | COVID-19 related mortality  and hospitalisation | Low risk; multivariable multinomial logistic regressions were conducted, including all covariates that were significant in the bivariable analysis. Adjustment was made for age, sex, race/ethnicity, smoking status, CCI score, time of COVID-19 diagnosis, BMI category, SBP (quartile), DBP (quartile), HDL level (quartile), and LDL level (quartile). Additional adjustments for HbA1c level, insulin use, metformin use, DPP-4 inhibitor use, GLP-1R agonist use, and SGLT2 inhibitor use in analyses of patients with type 2 diabetes. | Serious risk: prevalent user design, users of intervention were compared to non-user | Low risk; both cohorts were defined by using clear definition: A dispensed diabetes medication (unless the medication was metformin, a thiazolidinedione) | Low risk; ITT analysis conducted | Low risk: missing information on HbA1c levels, however they adjusted for CCI, this could be a proxy for severity of the diabetes. Note: Authors discuss missing information related to smoking status, although this is mentioned as an adjustment in table 2. | Moderate risk; outcomes of interest were proxy measures of COVID-19 severity; individuals were classified into three categories based on 30-day outcomes as indicated by site of care. The possibility of misclassification due to missing data was not ruled out. | Moderate risk; no a priori protocol, but there was no indication of selective reporting based on intervention, outcome, multiple analyses, or different subgroups | Serious risk of bias |
| Pérez-Belmonte, 2020 | COVID-19 related mortality, hospitalisation and other outcomes | Low risk; PS matching including following variables: age; gender; history of smoking, hypertension; dyslipidaemia; chronic kidney disease; cerebrovascular disease; chronic obstructive pulmonary disease; atrial fibrillation; coronary artery disease; heart failure; obesity; dementia; Barthel Index score; and Charlson Comorbidity Index score; treatment with angiotensin-converting enzyme inhibitor, angiotensin II receptor blocker, anticoagulant, and statin; admission blood glucose; serum creatinine; and transaminase  levels) | Serious risk: prevalent user design, users of intervention were compared to non-user | Low risk; both cohorts were defined: patients were grouped according to glucose-lowering drugs in monotherapy and in combination with metformin. Patients were match with a patient of another group receiving other  glucose-lowering drugs in a 1:1 manner | Low risk; ITT analysis conducted | Moderate risk: data provided about at-home glucose-lowering drugs did not include information on treatment adherence or treatment duration | Moderate risk: outcomes of interest were established as in-hospital death according to each at-home glucose-lowering drug in monotherapy or in dual therapy with metformin. Secondary outcomes were the following: first, a composite outcome including the need for ICU admission, invasive and non-invasive mechanical ventilation, or in-hospital death; second, in hospital complications; and third, a long hospital stay. Although these were not properly defined. | Moderate risk; no a priori protocol, but there was no indication of selective reporting based on intervention, outcome, multiple analyses, or different subgroups | Serious risk of bias |
| Ramos-Rincón, 2021 | COVID-19 related mortality | Low risk: A multivariate analysis was performed to control for confounding variables such as: demographics (age, sex, acquisition), body mass index, comorbidities and dependence (degree of dependence, Charlson Comorbidity Index, hypertension, dyslipidaemia, coronary disease, cerebrovascular disease, peripheral vascular disease, atrial fibrillation, heart failure, dementia, chronic lung disease, obesity, malignancy, moderate-to-severe renal disease), symptoms (dyspnoea), physical examination (oxygen saturation <90%, temperature 37.8°C, tachycardia, quick sequential organ failure assessment score ≥2), severity grade of COVID-19 disease, laboratory findings (neutrophils, lymphocytes, haemoglobin, platelet count, glucose, estimated glomerular filtration rate, lactate dehydrogenase, C-reactive protein, alanine aminotransferase), and treatment (metformin, DPP-4i, insulin, SGLT-2i, GLP-1ra, ASA, ACEI, ARB, and statin). | Serious risk: prevalent user design, users of intervention were compared to non-user | Moderate risk: Interventions are not well defined. Patients were grouped by preadmission cardiometabolic therapy and by no survivors and survivors. | Low risk; ITT analysis conducted | Moderate risk; Due to the fact that there were some missing values, variables  which were not recorded for >25% of patients were excluded from  the analysis. Is not clear where this patients are allocated, if in intervention or in comparator | Low risk; variable definitions were reported in manuscripts published by the  SEMI-COVID-19 Network | Moderate risk; no a priori protocol, but there was no indication of selective reporting based on intervention, outcome, multiple analyses, or different subgroups | Serious risk of bias |
| Hu, 2024 | Sepsis/septic shock | Moderate risk; PS matched based on baseline covariates such as age; adjusted for age, sex, comorbidities, and medications. No adjustment for: diabetes duration, HbA1c, BMI. | Serious risk: prevalent user design, users of intervention were compared to non-user | Low risk; both cohorts were defined: patients who received SGLT2I were defined as the SGLT2I cohort and matched with patients that did not receive SGLT2I | Low risk; ITT analysis conducted | Low risk: missing information on HbA1c levels, however they adjusted for DCSI | Low risk: outcome was well defined: Sepsis/septic shock is the main outcome of this study. The main outcome was defined as patients who received diagnoses in at least two outpatient claims or at least one inpatient claim. The secondary outcome was defined as patients who only received a diagnosis in at least one inpatient claim. Any error measuring the outcome is unrelated to the intervention status. | Moderate risk; no a priori protocol, but there was no indication of selective reporting based on intervention, outcome, multiple analyses, or different subgroups | Serious risk of bias |
| Tan, 2024 | Sepsis, pneumonia and COPD | Low risk: Negative binomial regression was used to adjust for age, sex, calendar year, time since diabetes diagnosis, intensity of GLD therapy, insulin use, medication burden, and IRSD. | Low risk; active comparator new user design | Low risk; both cohorts were defined: The date of the first dispensing of SGLT2i or  DPP4i was defined as the index date, a 1-year washout period (no dispensing of either  SGLT2i or DPP4i within a year before the index date) was used. | Low risk; ITT analysis conducted | Low risk: clinical information, such as renal function, glycaemic control (glycated haemoglobin levels), and body mass index. It is unlikely differentially for intervention vs comparator | Low risk: hypothesis-free testing of ~500 medication-outcome pairs in a single study | Moderate risk; no a priori protocol, but there was no indication of selective reporting based on intervention, outcome, multiple analyses, or different subgroups | Moderate risk of bias |
| Tsai, 2024 | Infectious keratitis | Low risk: PS matched was used by adjusting the  demographic, medical and disease factors | Serious risk: prevalent user design, users of intervention were compared to non-user. | Low risk; both cohorts were defined by the application of prescriptions via the international ATC codes | Low risk; ITT analysis conducted. | Moderate risk: missing information involving blood glucose level in T2DM, the glycated  haemoglobin level in T2DM, the exact etiology of  corneal diseases (i.e. trauma, dry eye related, bacterial  infection, fungal infection), the external eye  photography of corneal diseases, the fluorescein stain  result of corneal disease, the culture result of the  corneal disease if existed, the therapeutic outcome of  corneal disease and the recurrence of corneal disease. The analysis is unlikely to have removed the risk of bias arising from the missing data | Low risk: missing information involving the blood glucose level in T2DM, the glycated haemoglobin level in T2DM, the exact etiology of corneal diseases (i.e. trauma, dry eye related, bacterial infection, fungal infection), the external eye photography of corneal diseases, the fluorescein stain result of corneal disease, the culture result of the corneal disease if existed, the therapeutic outcome of corneal disease and the recurrence of corneal disease | Moderate risk; no a priori protocol, but there was no indication of selective reporting based on intervention, outcome, multiple analyses, or different subgroups | Serious risk of bias |

# Supplementary 7 – Sensitivity analyses

**A.**


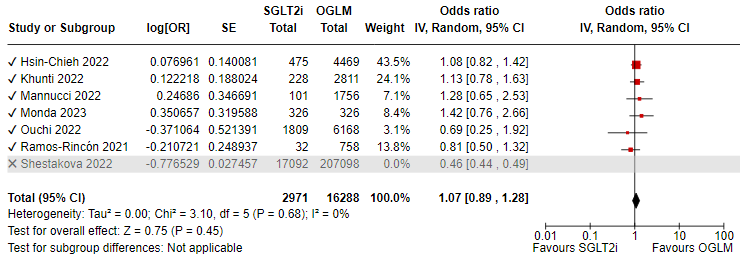


**B**.


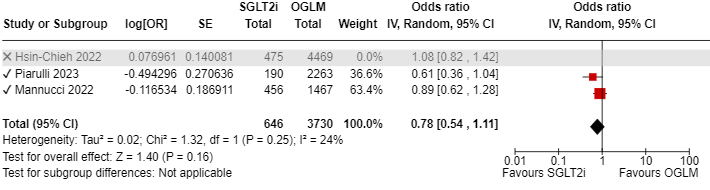


**C**.


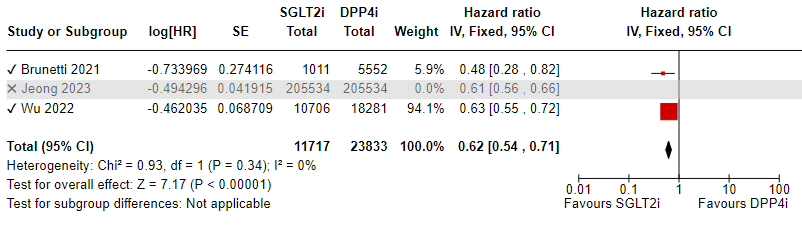


**D.**


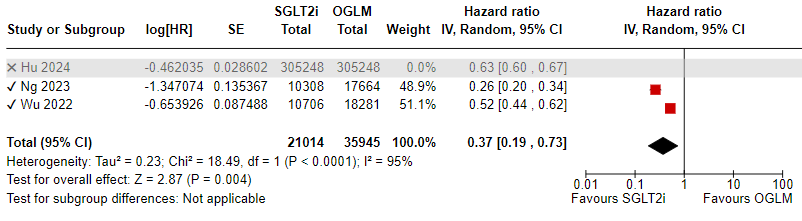


COVID-19 mortality (A), COVID-19 hospitalisation (B), pneumonia (C), and sepsis (D) in patients with type 2 diabetes compared with other glucose-lowering medication (OGLM) or to Dipeptidyl Peptidase-4 Inhibitors (DPP4i).

# Supplementary 8 – Funnel plots to judge the publication bias

A. B.


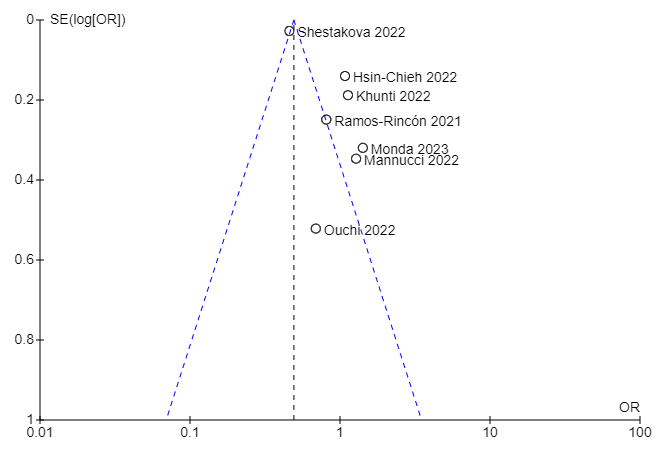

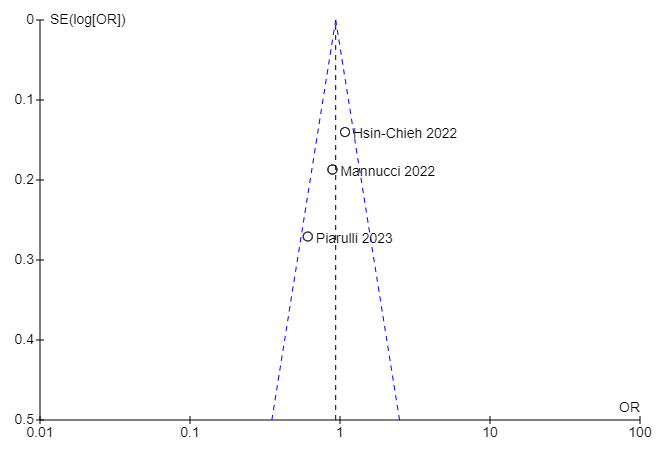


C. D. E.


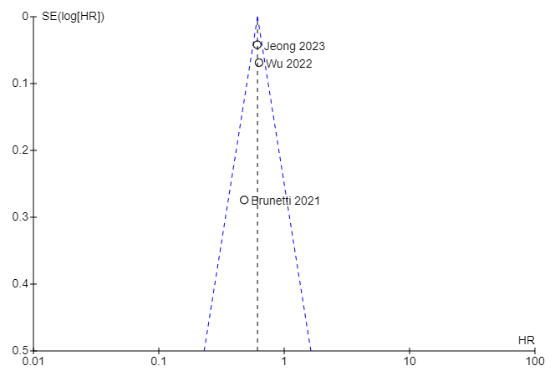

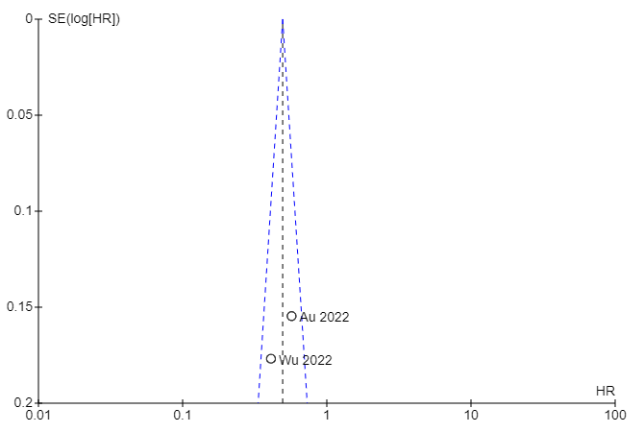

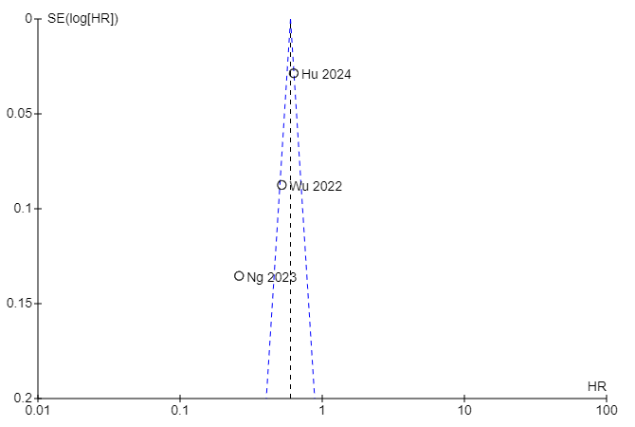


COVID-19 mortality (A), COVID-19 hospitalisation (B), pneumonia risk (C), pneumonia mortality (D), and sepsis (E).
